# When Photoelectrons Meet Gas Molecules: Determining the Role of Inelastic Scattering in Ambient Pressure X-ray Photoelectron Spectroscopy

**DOI:** 10.1021/acscentsci.4c01841

**Published:** 2024-12-20

**Authors:** Haoyi Li, Asmita Jana, Angel T. Garcia-Esparza, Xiang Li, Corey J. Kaminsky, Rebecca Hamlyn, Rajiv Ramanujam Prabhakar, Harry A. Atwater, Joel W. Ager, Dimosthenis Sokaras, Junko Yano, Ethan J. Crumlin

**Affiliations:** † Liquid Sunlight Alliance, Lawrence Berkeley National Laboratory, Berkeley, California 94720, United States; ‡ Chemical Sciences Division, Lawrence Berkeley National Laboratory, Berkeley, California 94720, United States; § Stanford Synchrotron Radiation Lightsource, SLAC National Accelerator Laboratory, Menlo Park, California 94025, United States; ∥ Molecular Biophysics and Integrated Bioimaging Division, Lawrence Berkeley National Laboratory, Berkeley, California 94720, United States; ⊥ Liquid Sunlight Alliance, California Institute of Technology, Pasadena, California 91125, United States; Δ Thomas J. Watson Laboratory of Applied Physics, California Institute of Technology, Pasadena, California 91125, United States; ¶ Department of Materials Science and Engineering, University of California Berkeley, Berkeley, California 94720, United States; ○ Advanced Light Source, Lawrence Berkeley National Laboratory, Berkeley, California 94720, United State

## Abstract

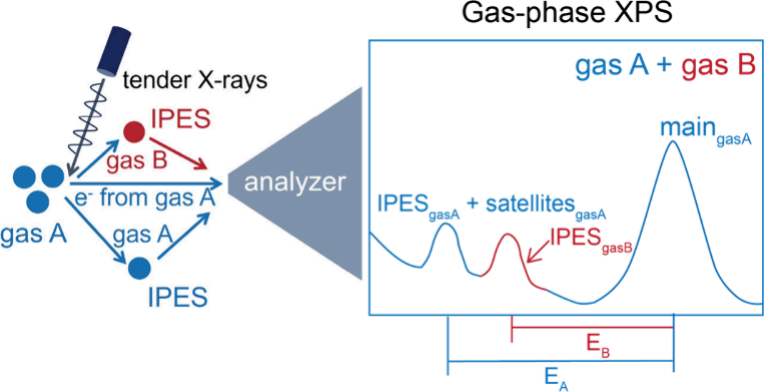

Inelastic photoelectron scattering (IPES) by gas molecules, a critical phenomenon observed in ambient pressure X-ray photoelectron spectroscopy (APXPS), complicates spectral interpretation due to kinetic energy loss in the primary spectrum and the appearance of additional features at higher binding energies. In this study, we systematically investigate IPES in various gas environments using APXPS, providing detailed insights into interactions between photoelectrons emitted from solid surfaces and surrounding gas molecules. Core-level XPS spectra of Au, Ag, Zn, and Cu metals were recorded over a wide kinetic energy range in the presence of CO_2_, N_2_, Ar, and H_2_ gases, demonstrating the universal nature of IPES across different systems. Additionally, we analyzed spectra of scattering effects induced by gas-phase interactions without metal solids. In two reported CO_2_-reduction systems (p-GaN/Au/Cu and p-Si/TaO_*x*_/Cu), we elucidated that IPES is independent of the composition, structure, or size of the solid materials. Using metal foil platforms, we further developed an analytical model to extract electron excitation cross sections of gas molecules. These findings enhance our understanding of IPES mechanisms and enable the predictions of IPES structures in other solid–gas systems, providing a valuable reference for future APXPS studies and improving the accuracy of spectral analysis in gas-rich catalytic interfaces.

## Abstract 2

Inelastic photoelectron scattering by gas molecules is comprehensively studied for ambient pressure X-ray photoelectron spectroscopy analyses, advancing spectral interpretation in gas-rich interfaces.

## Introduction

*In situ* and *operando* studies conducted under real-world conditions are crucial for understanding catalytic processes, where surface reactions play a pivotal role in performance.^1−3^ In this context, ambient pressure X-ray photoelectron spectroscopy (APXPS) stands out as a powerful analytical tool, providing fundamental insights into surface mechanisms.^4−6^*In situ* and *operando* measurements carried out at elevated pressures and temperatures via APXPS enable the determination of dynamic chemical states and surface evolutions, such as those involved in CO_2_ reduction and water oxidation, which demonstrate significance for designing more efficient catalysts and fine-tuning reaction conditions.^7−9^ The power of APXPS lies in its ability to capture real-time surface changes, but the analytical accuracy depends on the complexity of the XPS spectra. Typically, mechanistic insights in APXPS are largely derived from changes in the main photoemission lines, while Auger features are used to complement interpretation.^1,6^ However, satellite peaks, which appear adjacent to the core-level main peaks, also reveal essential information regarding the electronic structures and chemical transformations at material surfaces and interfaces.^10^ While these satellite peaks enrich the overall interpretation and allow for a comprehensive understanding of surface processes, their interpretations have largely been performed under ultrahigh-vacuum conditions where there are no bulk gas- or liquid-phase molecules that can interact with the photoelectrons.^1,11^

**Scheme 1:**
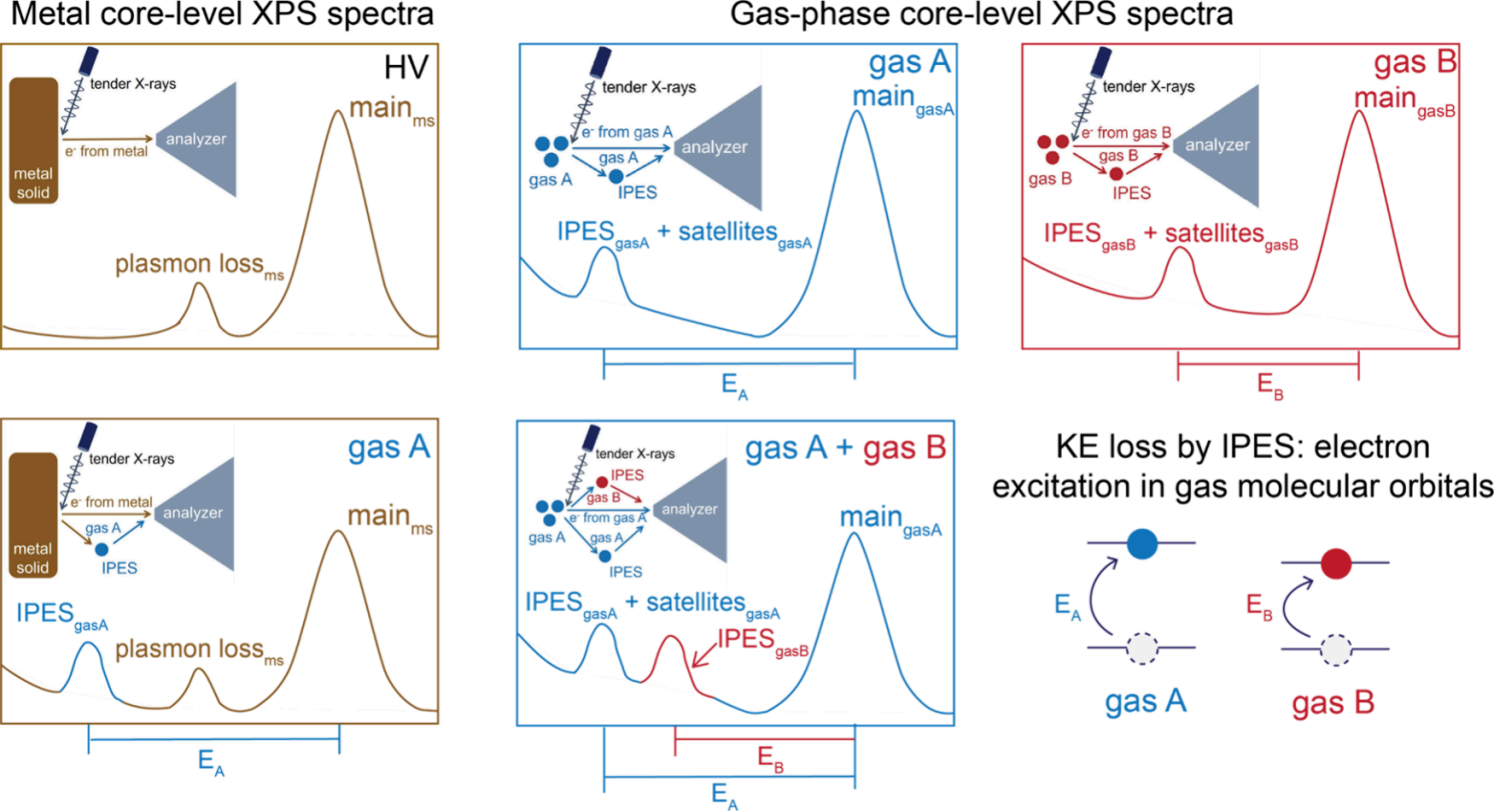
Schematic Illustration of the IPES in the APXPS System Highlighting its effects on the core-level XPS spectra of the metal solid (denoted as ms) and gas phases, including KE loss in the primary spectrum and the appearance of additional features at higher BEs. The insets exhibit the experimental configurations under the respective condition.

As the operating gas pressure increases, APXPS spectra become progressively more complex due to inelastic photoelectron scattering (IPES) induced by gas molecules.^12,13^ After photoelectrons are emitted from a solid surface or gas molecules, they travel hundreds of micrometers to reach the analyzer nozzle. In a gas-phase environment at Torr-level pressures, these photoelectrons collide with gas molecules during their traveling and thus lose kinetic energy (KE) through inelastic scattering. This scattering not only reduces the intensity of main peaks in metal and gas-phase XPS spectra, but also introduces additional spectral features at higher binding energies (BEs), complicating spectral deconvolution.^14^ Importantly, these additional peaks should not be misinterpreted as solid-state features; they arise from the inelastic scattering of photoelectrons emitted from the solid-state target at energies corresponding to the main XPS lines, and they interact with the surrounding gas. The KE losses during IPES resonate with electron excitations in the electronic structure of the gas molecules (Scheme 1).^15−18^ For a photoelectron contributing to an electron transition or ionization event within a gas molecule, it needs KEs ranging from tens to thousands of eV, exceeding the threshold required for the electron transitions or ionization of the gas. The first few transitions or ionization events, being the lowest energy processes, generally occur with relatively high probabilities. Each scattering event has an associated probability, and the intensity of the IPES structures is proportional to the probability. Thus, multiple scattering peaks become gradually less intense as the probabilities decrease.^13^ Consequently, scattering features resulting from photoelectrons undergoing only one scattering event, particularly those leading to one of the first few transitions, exhibit the highest intensities.

In this study, we provide a detailed analysis of IPES using APXPS with tender X-rays, aiming to clarify its effects on XPS spectra and offer a reliable reference for future studies. We present the core-level spectra of polycrystalline metal foils (Au, Ag, Zn, Cu) across an extended BE range (approximately 20 eV above the main peaks) collected under exposure to various gases, namely CO_2_, N_2_, Ar, and H_2_. By comparing these spectra with the gas-phase spectra collected without the presence of photoelectrons generated by solid metals, we isolated the IPES structures that arise purely from gas-phase interactions, which allows us to highlight the distinct scattering features of the photoelectrons. To prove the universality and impacts of IPES in the APXPS system, we examined two reported CO_2_ reduction systems (p-GaN/Au/Cu^19^ and p-Si/TaO_*x*_/Cu^20^), demonstrating that the IPES structures are independent of the composition, structure, and size of the solid materials. We further identified a linear relationship of the intensity ratio of the IPES peak(s) to the main peak(s) as a function of gas pressure, which was supported by an analytical model, which can serve as a vital resource to predict and assess IPES structures for other solids and gases. Based on the analytical model, we derived a new strategy to measure electronic excitation cross sections of the gases. The findings advance both the fundamental understanding and practical handling of IPES, improving the accuracy of APXPS analyses.

## Results and Discussion

**Figure 1:**
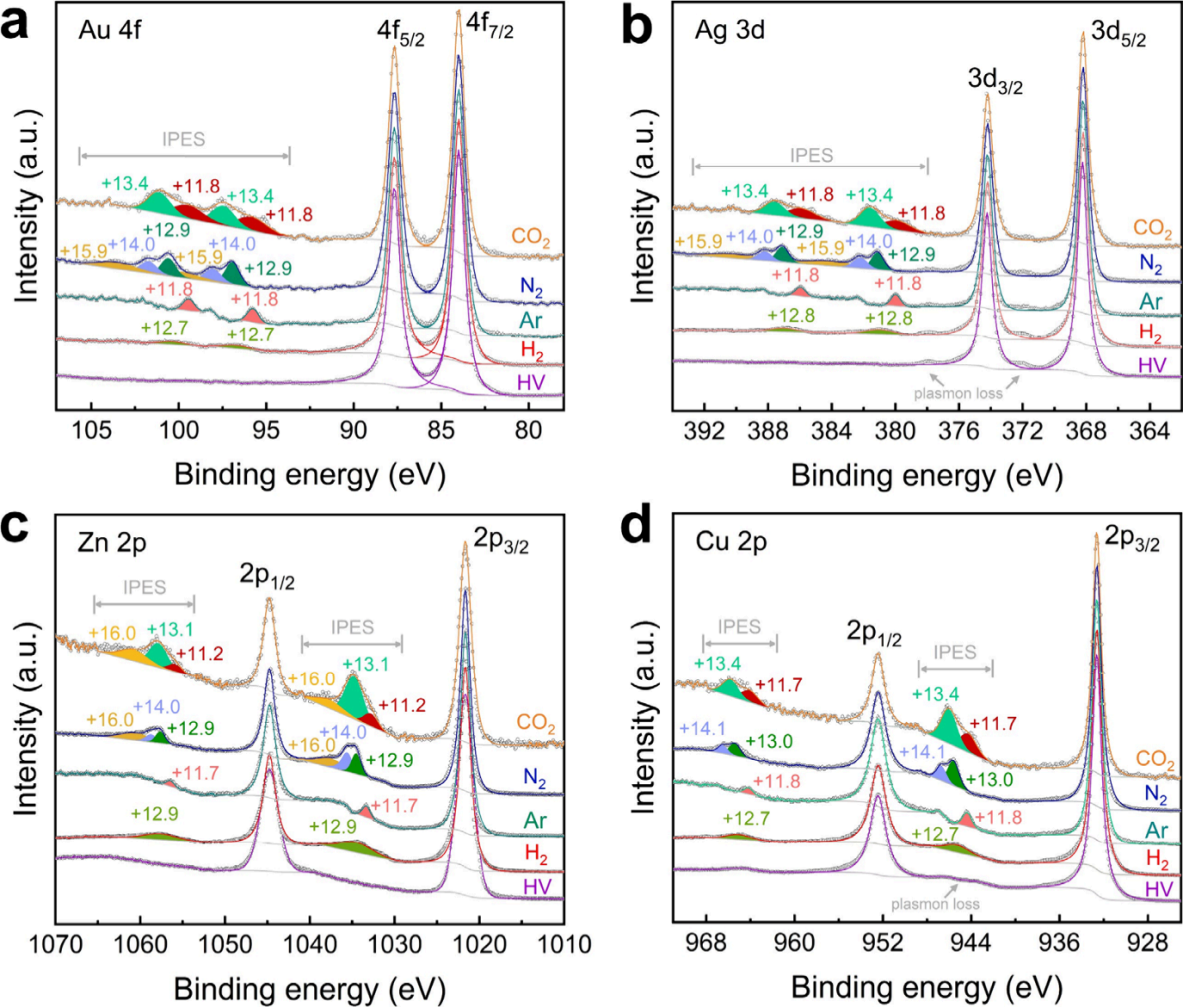
IPES features induced by the surrounding gas molecules in the core-level spectra of metal solids. Deconvoluted IPES structures (solid-colored areas) are identified by the differences in BE compared to that of the main peaks in the (a) Au 4f, (b) Ag 3d, (c) Zn 2p, and (d) Cu 2p regions collected at room temperature under HV; 5 Torr of H_2_; and 15 Torr of CO_2_, N_2_, and Ar conditions. The differences in BEs between each pair of IPES structures and main lines are indicated above the corresponding IPES structures.

We utilized polycrystalline transition metal foils (measuring 1 cm in length, 1 cm in width, and 0.25 mm in thickness), namely Au, Ag, Zn, and Cu, for APXPS analysis under various conditions. Detailed information regarding the sample preparation processes is provided in the Supporting Information. APXPS experiments were conducted using a photon energy of 4 keV at room temperature. We present the core-level spectral deconvolution in the Au 4f, Ag 3d, Zn 2p, and Cu 2p regions, with collection of the extended BE range to approximately 20 eV beyond the main peaks (Figure 1).

These spectra were collected under various conditions of high vacuum (denoted as HV, pressure below 10^–4^ Torr); 5 Torr of H_2_; or 15 Torr of CO_2_, N_2_, or Ar. Under HV condition, no significant peaks were apparent at BEs greater than that of the main peaks, and the main peaks indicated the predominant metallic states on the surface of the metal solids. For the Ag and Cu metal foils, we observed weak satellite peaks located at +3.9 and +13.1 eV above the BE positions of the main peaks in the Ag 3d and Cu 2p regions, respectively, under HV condition. These features arise from plasmon loss on the metal surfaces.^21,22^

IPES were investigated in several gas environments including CO_2_, N_2_, Ar, and H_2_. Given their widespread utilization in catalytic reactions, we selected CO_2_ and H_2_ as representative gas molecules, allowing for interpretation of spectra collected from a range of reaction dynamics.^2,3,9,19^ H_2_, being the simplest gas molecule, is notoriously difficult to detect by XPS.^23^ However, in the metal core-level spectra, we were able to observe excitations within the electronic structure of H_2_, thus identifying the feature of H_2_ molecules through XPS (Figure 1). In parallel, we utilized inert gases (N_2_ and Ar) in our APXPS measurements to rule out any potential changes in electronic structure or surface chemistry of the metal foils induced by reactive gases, providing solid evidence for IPES.^24,25^ Moreover, by comparing Ar with other gases, we distinguished the IPES structures under atomic and molecular gas conditions.

**Figure 2:**
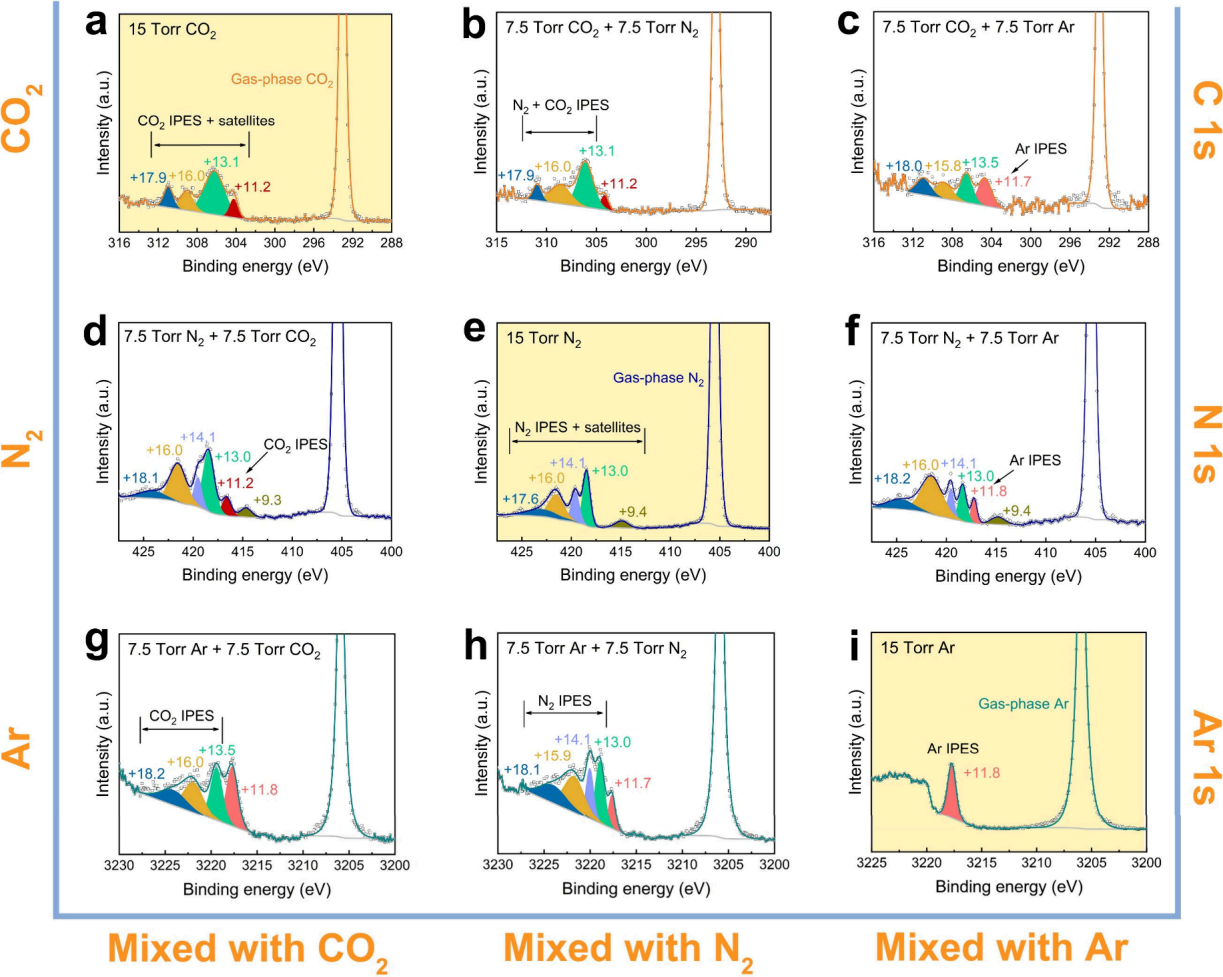
Gas-phase XPS spectra without metal solids, illustrating the gas-dependent interactions between photoelectrons and gas molecules. Deconvoluted IPES structures in the (a), (b), (c) C 1s, (d), (e), (f) N 1s, and (g), (h), (i) Ar 1s regions are identified by differences in BE from the main peaks, collected under pure gases and pairwise mixtures of CO_2_, N_2_, and Ar at a total pressure of 15 Torr. The yellow background highlights the spectra collected under single-gas conditions, distinguishing them from those collected under pairwise-gas conditions.

Upon dosing gases into the APXPS system, IPES structures appear at greater BEs than those of the main peaks, with values ranging from approximately +11 to +17 eV higher. These differences in BE between the IPES and main peaks are indicated in Figure 1 and can be considered as the KE losses of photoelectrons excited from the metal surface. Notably, the deconvoluted IPES structures in all metal core-level spectra exhibited consistent BEs and line shapes in the same gas condition, which changed when another type of gas was used. Thus, the IPES structures we observed indicate the interaction between the excited photoelectrons and gas molecules in the APXPS system.^12−14^ Based on this gas-specific phenomenon, we further analyzed single and pairwise gas-phase spectra (without metal solids) at a total pressure of 15 Torr to determine the origins of the IPES in particular gas environments. The spectra recorded in single gas environments (Figure 2a,e,i) highlight inherent IPES structures induced by CO_2_, N_2_, and Ar. The BE differences from the main peaks and varied line shapes of the IPES structures in the metal core-level spectra (Figure 1) are well corroborated by the scattering features in the gas-phase spectra collected under the same conditions (Figure 2 and Scheme 1).

However, satellite peaks from gas-phase photoemission lines can also appear in a BE range similar to that of the IPES structures, leading to potential overlap of the two types of peaks. To distinguish IPES structures from satellite peaks, we recorded gas-phase spectra under pairwise gas conditions, enabling clear differentiation of IPES structures from satellite peaks. In the case of pure Ar, a single peak appears at 11.8 eV higher than the main peak in both the Ar 1s and Ar 2p regions (Figure 2i and S1a). This peak arises from photoelectrons scattered by Ar atoms, corresponding to an electron transition from the 3p to 4s orbital of Ar (11.8 eV).^12,26^ It has been reported that Ar satellite peaks are located over 20 eV above the main peaks of the gas-phase specta.^27,28^ In the mixed environments of CO_2_-Ar and N_2_-Ar, the same +11.8 eV peak is observed in the C 1s and N 1s regions (Figures 2c,f and S1d), but it disappears in pure CO_2_ and N_2_ atmospheres (Figure 2a,e), confirming that this is an IPES structure unique to Ar, rather than a satellite peak. On the other hand, the scattering features at higher BEs recorded in pure CO_2_ and N_2_ environments likely represent a combination of both IPES and gas satellite peaks (Figures 2a,e and S1f). To further isolate the IPES structures of N_2_ and CO_2_, we compared spectra collected in mixed and single gas environments. For example, the Ar 1s spectrum obtained in the mixed Ar-N_2_ condition includes both IPES structures of N_2_ and Ar but excludes satellite peaks of N_2_ gas. This principle also enables IPES structure identification for other gases. According to the observations under the CO_2_-N_2_ and Ar-N_2_ mixture environments (Figures 2b,h and S1b,e), peaks at +13.0, +14.1, +16.0, and +17.6 eV above the main peak of gas-phase N_2_ (Figure 2e) are identified as characteristic IPES structures for N_2_.^29−31^ Similarly, for CO_2_, peaks at +11.2, +13.1, +16.0, and +17.9 eV greater than the main peak of gas-phase CO_2_ (Figure 2i) are assigned as IPES structures based on the features measured under the mixed conditions of N_2_-CO_2_ and Ar-CO_2_ (Figures 2d,g and S1c).^32,33^

IPES structures in the metal core-level spectra represent the scattering events with the highest probabilities, while the elevated background and spin–orbit splitting obscure the lower-probability scattering features, especially for CO_2_ and N_2_ (Figures 1 and 2a,e). The individual IPES structures highlighted in Figures 1 and 2 by solid colors were determined based on the BE values previously reported, which were used as the XPS curve-fitting constraints. This approach ensures that our assignments are guided by established reference results. The use of reported BEs as benchmarks provides a reliable foundation for identifying and deconvoluting these IPES structures. The BE values obtained from our experiments are comparable with those previously reported (Table S1), which demonstrates the consistency of our assignments and provides transparency regarding the alignments between our results and established references. The distinctive energy losses of +11.2 eV and +13.1 eV beyond the CO_2_ main peak correspond to the excitation states of ^1^Σ_u_^+^ (which is also likely to be ^3^Σ_u_^–^, ^1^Π_g_, or ^3,1^Π_u_), and the first ionization transition state of CO_2_^+^^2^Π_g_, respectively.^15,16,34−36^ In the case of N_2_, the IPES structures at +13.0, +14.1 eV, and +16.0 eV compared to the main peak of N_2_ can be corelated to two excitation states of ^2^Π_g_ and ^2^Σ_g_^+^,^37−42^ respectively. Additionally, several excitation states (C^1^Π_u_, B^1^Σ_g_^+^, c^3^Π_u_, a^3^Σ_g_^+^, E(F)^1^Σ_g_^+^) within the H_2_ molecular orbitals inform the IPES structures observed for H_2_ molecules within the metal core-level regions.^43−47^ The assignments of all IPES structures are summarized in Table S1. We categorize the observed peaks by their corresponding excitation states, each of which involves groups of closely spaced excitation energies rather than individual transitions. This approach acknowledges the inherent challenge of resolving every individual excitation energy within a single excitation state due to the spectral broadening and the overlapping of peaks. Thus, electronic transitions in gas molecules are measurable via IPES, providing valuable insight into gas-phase interactions in the APXPS system.

To address the concerns about beam-induced effects on Cu metal foil surfaces, which are highly sensitive to X-ray exposure,^3,48^ we conducted additional control experiments. The CO_2_ dosing process was repeated with and without X-ray exposure, and Cu 2p_3/2_ spectra were collected in both cases. The IPES structures remained in CO_2_ under both conditions but disappeared under HV condition (Figure S2), confirming that beam-induced effects on the Cu surface were negligible during our measurements. Thus, we conclude that the features observed at higher BE positions relative to the main peaks on the Cu metal foil were indeed caused by IPES, as expected. The core-level spectra of transition metals collected under various gas conditions can serve as valuable references for future APXPS studies, helping to prevent the misinterpretation of XPS spectra due to IPES artifacts.

**Figure 3:**
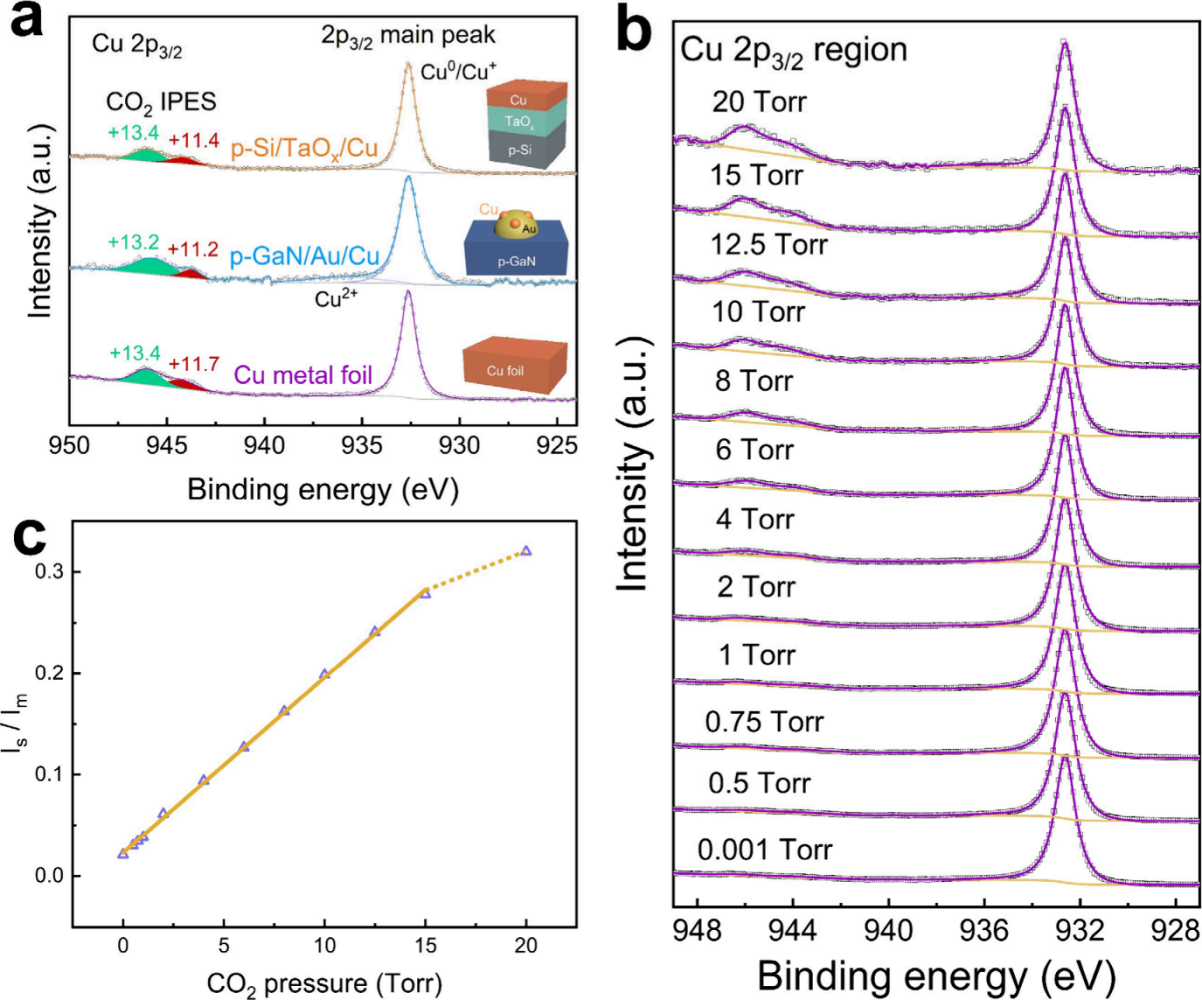
Independence of IPES on the composition, morphology, and size of the solid materials, as well as pressure-dependent IPES features induced by gas molecules. (a) Deconvoluted Cu 2p_3/2_ regions collected under 15 Torr of CO_2_ on p-GaN/Au/Cu, p-Si/TaO_*x*_/Cu and polycrystalline Cu metal foil. The inset schemes show the structure and composition of the three materials systems. (b) Cu 2p_3/2_ spectra collected on the Cu metal foil with increasing CO_2_ pressure. (c) Calculated area ratio between IPES and main peaks (*I*_s_/*I*_m_) as a function of CO_2_ pressure. The linear fitting curve was obtained between 0.001 and 15 Torr of CO_2_. The corresponding slope and intercept are 0.017 ± 1.57 × 10^–4^ Torr^–1^ and 0.023 ± 1.16 × 10^–3^, respectively, and the coefficient of determination is 0.999.

To further demonstrate the universality of IPES, we extended our investigation to recently developed CO_2_ reduction systems, p-GaN/Au/Cu^19^ and p-Si/TaO_*x*_/Cu,^20^ measured under 15 Torr of CO_2_. As shown in Figure 3a, the p-GaN/Au/Cu system features small Cu nanoparticles (5 nm) supported by larger Au nanoparticles (40 nm) on a p-GaN substrate. The Cu nanoparticles contained a small amount of Cu^2+^, likely resulting from air exposure.^19^ In contrast, the p-Si/TaO_*x*_/Cu system exhibits a layer-by-layer thin-film structure, with a 10 nm Cu thin film deposited on a 40 nm TaO_*x*_ thin film supported by a p-Si substrate. Similar IPES structures were observed in all three Cu-based platforms, indicating that IPES is independent of the sample composition, morphology, and size of the solid materials.

The positions of the IPES structures showed a shift to higher BEs by less than 0.6 eV compared to theoretical calculations.^12,14^ One reason for this shift is the larger full width at half-maximum of the IPES structures caused by higher gas pressures, leading to peak overlapping, including additional scattering features, which contribute to the increased BE. Another reason is that experimental results involve both electronic and vibrational excitations of gas molecules, whereas theoretical BE values account only for electronic excitations, potentially leading to additional peaks in the experimental spectra.

Using polycrystalline Cu metal foil as a model platform, we varied the pressure of CO_2_ from 0.001 to 20 Torr, which is pertinent to the studies on the interfacial chemical states of Cu catalysts in CO_2_ atmosphere via APXPS. The corresponding XPS spectra (Figure 3b) collected under increasing CO_2_ pressures exhibited similar IPES structures in terms of BEs and line shape. Intriguingly, the intensity ratio of the IPES to the main peaks showed a linear correlation with CO_2_ pressure below 15 Torr (Figure 3c and Table S2), suggesting a higher likelihood of IPES created by the excited photoelectrons interacting with gas molecules at higher pressures. However, as the pressure further increased to 20 Torr, the slope decreased (the dashed line in Figure 3c), implying that the apparently increased collisions of photoelectrons with the surrounding gas molecules at a higher pressure may lead to the overall attenuation of spectral features. Multiscattering probability of photoelectrons is also increased at 20 Torr compared to that at lower pressures, which results in the intensity decrease of the IPES structures.

Using the main peak from the metal with gas scattering features, we further investigated the peaks of the first few transition events via an analytical model. These scattering peaks exhibit BE differences aligning well with the most likely energy transitions observed in the gas molecules. Every time a gas molecule inelastically scatters a photoelectron, the photoelectron loses some of its KE. Thus, only the photoelectrons originating from the metal region that have never been inelastically scattered contribute to the metal main peak intensity. Similarly, only the photoelectrons originating from the metal region that are inelastically scattered once and lead to the first few transition events in the gas contribute to the first few scattering peak intensities.^15,26,37,44^ The intensity ratio of the IPES to the main peak is given by eq 1.
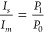
1where *P*_*0*_ and *P*_*1*_ are the probabilities of an electron scattering 0 and 1 times, respectively. These probabilities contain information on the transition energies and the initial KE of the photoelectron.^49^

Assuming the thickness between the sample and detector (*t*) is divided into *N* infinitesimally small sections, with a thickness of *t*/*N*, the photoelectron undergoes inelastic scattering *t*/(*N*λ), where λ represents the inelastic mean free path. Subsequently, the probability of n scattering events can be described by the binomial distribution in eq 2.^13^
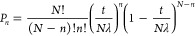
2

The probability of no scattering and one scattering event are given by eqs 3 and 4, respectively.
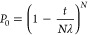
3
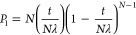
4

By combining eqs 3 and 4 to derive the ratios, we obtain eq 5, which determines the dependence of intensity ratios on the physical characteristics of the system. Here, *p*, *T*, R and σ denote the pressure, temperature, gas constant, and cross section, respectively.
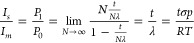
5

In our experiments, we assessed the intensity ratios of the IPES to the main peaks, varying pressures from 0.001 to 15 Torr at 298 K (Figure 3c), with a constant detector-to-sample distance of 0.35 mm. We considered the cross section as the one corresponding to the specific electronic excitation referred to as the electronic excitation cross section. Linear fitting of the intensity ratios to CO_2_ pressure yielded a slope of 0.017 Torr^–1^ with an R^2^ of 99.9%. Similar fits were generated for other gas/metal pairs, as displayed in Figure S3 and Tables S2 and S3. Their slopes and R^2^ values (as the uncertainties of the slopes) are detailed in Table S4. The slope, as indicated by eq 5, equals *t*σ/*RT*. Since *t*, *R*, and *T* are constants across all gas/metal pairs, the difference in the slopes depends solely on the electronic excitation cross section, σ.

**Figure 4:**
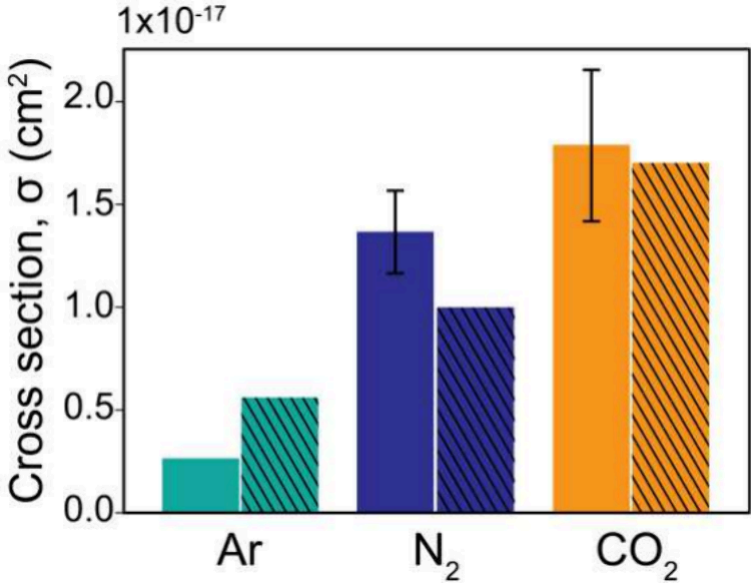
Comparison of electronic excitation cross sections (σ) obtained in this work (solid color bars) with those from previous studies (solid color bars with diagonal lines) for Ar,^50^ N_2_,^18^ and CO_2_.^51^ The error bars represent the standard deviations of all the electronic excitation cross sections for the gases measured in this work using the metal solids. For Ar, the error bar is not visible, as the slopes for the intensity ratio of the IPES to the main peaks in the metal core-level spectra as a function of gas pressure were found to be identical.

Based on the slopes of other gas/metal pairs (Figure S3 and Table S4), we calculated the average electronic excitation cross sections (σ) for Ar, N_2_, and CO_2_, which are comparable with the values obtained using other experimental techniques^18,50,51^ for photoelectron KEs between 2800 and 4000 eV (Figure 4 and Table S5). While the electronic excitation cross sections for N_2_ and CO_2_ align well with those in the existing references, the value for Ar shows a minor discrepancy. This difference arises because our calculated electronic excitation cross section corresponds to the first few electronic transitions in the gases, whereas the values in the references represent the sum of all possible electronic transitions. Despite these differences, we used the literature values as a benchmark to ensure that the order of magnitude of our calculated electronic excitation cross sections is reasonable. Moreover, compared to monatomic Ar, there are more possible excitation pathways for N_2_ and CO_2_ due to their abundant bonding and antibonding molecular orbitals, providing more electronic states for electrons that can be excited by incoming energy. This increases the electronic excitation cross sections of N_2_ and CO_2_ compared to Ar.^16−18,34,50^

As a result, in addition to identifying IPES structures, our analytical method accurately captures the correlation between the intensity ratio of the IPES to the main peaks, allowing for the determination of the electronic excitation cross sections of gases. Notably, this work introduces a novel strategy to obtain the electronic excitation cross sections of gas molecules, providing valuable insights for the APXPS community. By incorporating parameters such as initial transition energies, electronic excitation cross sections, incident photon energy, detector-to-sample distance, and temperature, our model enables further exploration of the intensity ratio between IPES and main peaks across a variety of solid samples and gases, highlighting its broad applicability. Moreover, by using this model, peaks resulting from gas scattering can be effectively corrected, yielding increasingly accurate predictions of chemical changes in future studies.

## Conclusions

In this study, we evaluated the IPES in APXPS measurements with tender X-rays. Under ambient pressure conditions, distinctive scattering features indicative of this phenomenon were noticed in the core-level spectra collected from polycrystalline metal foils in the gas atmosphere, contrasting with spectra obtained under HV conditions. These findings serve as valuable references for the APXPS community. We determined the gas dependency and universality of IPES structures, providing insights into the intrinsic nature of this prevalent phenomenon. Using polycrystalline metal foils, we established the scope of IPES at practical operation conditions with an analytic model. By correlating the intensity ratio of the IPES to the main peaks with the probabilities of photoelectron excitation events, we demonstrated the measurable electronic excitation cross sections of the gases via IPES in APXPS measurements, which provides novel information for studies on electron transitions. Furthermore, the IPES structures in the XPS spectra can be also used to measure the gas pressure and calibrate the electron analyzer, further enriching the function of IPES. These comprehensive findings enhance our fundamental understanding of IPES in APXPS analyses, providing crucial insights into the emergence of scattering features and facilitating precise spectrum interpretation.

## Supporting Information Available

Detailed APXPS experimental methods, preparation methods of samples, additional XPS spectra and analysis (PDF)
Transparent Peer Review report available (PDF)
