# Supplementary material for: When Photoelectrons Meet Gas Molecules: Determining the Role of Inelastic Scattering in Ambient Pressure X-ray Photoelectron Spectroscopy
Source: ACS Cent Sci. 2024 Dec 20;11(1):98–106. doi: 10.1021/acscentsci.4c01841 (PMC11758507; doi:10.1021/acscentsci.4c01841)
Supplement: Supplementary file 1 — oc4c01841_si_001.pdf [file oc4c01841_si_001.pdf]

Supporting Information for

When Photoelectrons Meet Gas Molecules: Determining the Role of Inelastic Scattering in Ambient Pressure X-ray Photoelectron Spectroscopy

Haoyi Li[†,ᵀ], Asmita Jana[ᵀ], Angel T. Garcia-Esparza[†,‡], Xiang Li[†,‡], Corey J. Kaminsky[∥], Rebecca Hamlyn[ᵀ], Rajiv Ramanujam Prabhakar[†,ᵀ], Harry A. Atwater[ᴨ,Δ], Joel W. Ager[†,ᵀ,¶], Dimosthenis Sokaras[‡], Junko Yano[†,∥,*], Ethan J. Crumlin[ᵀ,§,*]

[†]Liquid Sunlight Alliance, Lawrence Berkeley National Laboratory, Berkeley, California, 94720, United States.

[ᵀ]Chemical Sciences Division, Lawrence Berkeley National Laboratory, Berkeley, California, 94720, United States.

[‡]Stanford Synchrotron Radiation Lightsource, SLAC National Accelerator Laboratory, Menlo Park, California, 94025, United States.

[∥]Molecular Biophysics and Integrated Bioimaging Division, Lawrence Berkeley National Laboratory, Berkeley, California, 94720, United States.

[ᴨ]Liquid Sunlight Alliance, California Institute of Technology, Pasadena, California, 91125, United States.

[Δ]Thomas J. Watson Laboratory of Applied Physics, California Institute of Technology, Pasadena, California, 91125, United States.

[¶]Department of Materials Science and Engineering, University of California Berkeley, Berkeley, California, 94720, United States.

[§]Advanced Light Source, Lawrence Berkeley National Laboratory, Berkeley, California, 94720, United State.

Experimental Section

Ambient pressure X-ray photoelectron spectroscopy (APXPS) experiments

All APXPS experiments were conducted at the beamline 9.3.1 of Advanced Light Source, Lawrence Berkeley National Laboratory. A Si(111) double crystal monochromator was utilized to provide an energy range between 2.0 and 6.0 keV, referred to the "tender" X-ray range. The pass energy of the Scienta analyzer (R4000 HiPP-2) was set to 100 eV. A step size of 100 meV and a dwell time of 250 ms were employed. The sample holder was directly transferred from the ambient environment to the main chamber of the end-station for experiments and electrically connected to a multi-axis manipulator. Both the sample surface and the analyzer front cone were grounded in our APXPS system. The incidence angle between the incoming photons and the sample surface was set at 15 degrees, with a distance of 0.35 mm between the sample surface and the analyzer cone. The beamline is separated from the main chamber by a 2 mm × 2 mm × 100 nm (length × width × thickness) Si_3N_4 window, which maintains an ultrahigh vacuum (UHV) condition ranging from 10^{-9} to 10^{-7} Torr and protects the beamline from contamination of optical elements. The pressure range in the main chamber for operando experiments varies from high vacuum (HV, 10^{-4} Torr) to 20 Torr, while the corresponding detection range in the analyzer is from 10^{-9} to 10^{-7}. The pressure value was obtained by 626D Unheated Absolute Baratron® Capacitance Manometers from MKS Instruments, which shows the error below 0.25%. Optimization of photon energy intensity was achieved by the Au 4f spectrum at the focal point (0.35 mm away from the front cone) with the desired photon energy. For each condition, samples were equilibrated, and time-dependent scans of metal core-level spectra were conducted to track changes until the spectra stabilized. Subsequently, high-resolution measurements were performed for each spectrum.

The spectra deconvolution was performed using CasaXPS version 2.3.19PR1.0 (Casa Software Ltd, Teignmouth, UK). The binding energy (BE) calibration was achieved utilizing the Au $4f_{7/2}$ peak (BE = 84.0 eV as a reference). A symmetrical Voigt function, with a Gaussian/Lorentzian ratio ranging from 100/0 to 5/95, was employed to fit the spectra. Shirley background subtraction was applied, except for inelastic photoelectron scattering (IPES) peaks, which underwent linear background subtraction.

Sample Preparation

Polycrystalline metal foils. The original polycrystalline metal foils, including Au, Ag, Zn, Cu, and Ni (with a thickness of 0.25 mm and a purity of 99.98% trace metals basis), were provided by Sigma-Aldrich. Prior to the APXPS experiments, the samples underwent a cleaning procedure as follows: A uniform piece (1×1 cm) of the original foil was tailored and placed into a clean beaker. Subsequently, the sample was washed sequentially with water, ethanol, acetone, and water, each step being repeated at least three times. Ultrasonication was applied to the sample in each solvent

for a minimum of 30 minutes. Following natural drying in a nitrogen atmosphere, the sample surface was thoroughly polished and affixed to a stainless-steel sample holder. The sample holder was then transferred to the main chamber at BL 9.3.1, and the main chamber was evacuated overnight in preparation for the APXPS experiments.

p-GaN/Au/Cu. The synthesis of p-GaN/Au/Cu was recently developed[1]. p-GaN/sapphire substrates (with a c-axis 0001 orientation) were procured from University Wafer. The GaN layer is approximately 5 µm thick, consisting of a top layer of 2-2.5 µm p-GaN with Mg doping concentration exceeding 6×10^{16} cm^{-3} and an underlying layer of 2-2.5 µm undoped GaN. The substrates were cut into 1×1 cm pieces and cleaned through sequential ultrasonication in acetone, isopropyl alcohol, and nanopure water, each for 5 minutes. Subsequently, the samples underwent a 30-minute immersion in freshly prepared aqua regia to eliminate impurities and native oxide on the GaN surface. Following this, the samples were rinsed thoroughly with nanopure water and dried under N_2. Next, a 5-nm thick Au thin film was deposited onto commercial p-type GaN/sapphire substrates (c-axis 0001 orientation, 4.5 µm thick GaN supplied from Pam-Xiamen) via electron-beam physical vapor deposition (System 02520, Angstrom Engineering) at a base pressure of approximately 1 x 10^{-7} Torr, with a deposition rate of 1.0 Å/s. Subsequent to this, an annealing process was conducted on the p-GaN/Au film in ambient air at 500 °C for 3 hours, which transformed the Au film into Au nanoparticles with strong adhesion to the p-GaN surface. Following the annealing, a 1-nm thick Cu film was deposited via electron-beam deposition onto the p-GaN/Au at a base pressure of approximately 1 x 10^{-7} Torr, with a deposition rate of 1.0 Å/s. Following these steps, the p-GaN/Au/Cu structure was successfully prepared.

p-Si/TaO$_x$/Cu. The standard procedure for preparing p-Si/TaO$_x$/Cu was recently documented[2]. The process involved sequential cleaning of Si wafers by ultrasonication in acetone (10 min), soap water (10 min), deionized water (10 min), and iso-propyl alcohol (10 min). Subsequently, a 1% HF solution was applied to etch and remove the native oxide layer from the Si wafers. Following HF treatment, the Si wafers were promptly loaded into the sputtering chamber to fabricate the p-Silicon photocathodes. The next step involved depositing TaO$_x$ onto the p-Si substrate using an AJA International ATC Orion 5 sputtering tool. A pure Ta sputtering target was utilized with an RF power of 150 W. Before deposition, the Ta target underwent pre-sputtering for 30 minutes to eliminate any oxide layer. Deposition occurred at a pressure of 3 mTorr in the atmosphere of Ar and O_2 mixture, with a 97:3 ratio of Ar to O_2. The deposition duration was 3 minutes, resulting in a TaO$_x$ thickness of 180 nm. Finally, Cu was sputtered onto the TaO$_x$ layer using pure Cu metal targets and an RF power of 100 W. Sputtering took place at a pressure of 3 mTorr under an Ar atmosphere. The sputtering time for Cu varied from 2 to 10 seconds.

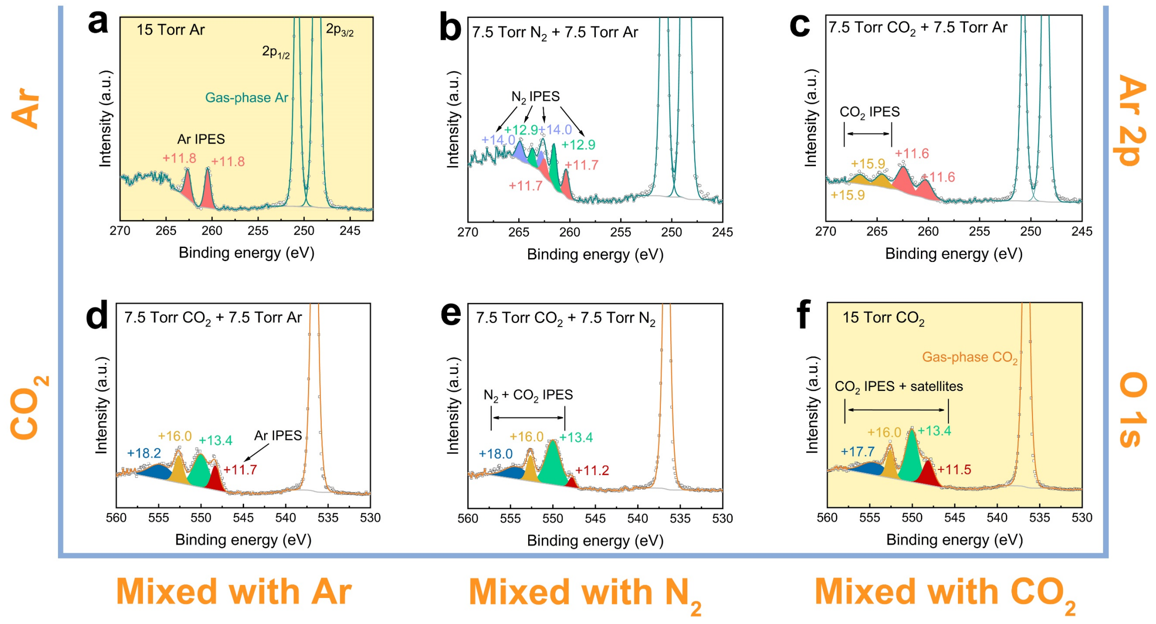

Figure S1. Gas-phase XPS spectra without metal foil samples illustrate gas-dependent interaction between photoelectrons and gas molecules. Deconvoluted scattering signatures in (a), (b), (c) Ar 2p and (d), (e), (f) O 1s regions are identified by the differences in binding energy from the main peaks collected under pure and pairwise mixtures of Ar, N_2, and CO_2 at the total pressure of 15 Torr, respectively. The yellow background highlights the spectra collected under single-gas conditions, distinguishing them from those collected under pairwise-gas conditions.

Table S1. The comparison of the BE values obtained from our experiments with those reported in the references. Peak assignments were exhibited as the possible excitation states shown in the references, especially those in the molecular orbitals of H_2, CO_2 and N_2. Broad fitting peaks were used in the metal and gas-phase core-level spectra to fit the IPES peaks in **Figure 1**, **2** and S1, which may account for multiple transitions that occur with closely spaced transition energies, as shown in the electron energy loss spectroscopy (EELS) for the gases in the references.

Gas	Our BEs (eV)	Literature BEs (eV)	Peak Assignment	References
Ar	11.6-11.8	11.6-11.8	Electron transition from the $3p^6$ to $3p^5 4s^1$ orbital	1. *J. Electron Spectrosc.* 2019, 232, 111-120; 2. *J. Chem. Phys.* 1968, 48, 5066-5096.
H_2	12.7-12.9	11.2-14.6	Electron excitation from X $^1\Sigma_g^+$ to B $^1\Sigma_g^+$	1. *J. Phys. B: At. Mol. Opt. Phys.* 2002, 35, 4695; 2. *J. Phys. B: At. Mol. Opt. Phys.* 2017, 50, 4075; 3. *J. Mol. Spectrosc.* 1969, 32, 39-53; 4. *J. Chem. Phys.* 1966, 44, 440-441; 5. *J. Mol. Spectrosc.* 1972, 41, 425-486.
		11.8-14.7	Electron excitation from X $^1\Sigma_g^+$ to c $^3\Pi_u$	
		11.8-14.7	Electron excitation from X $^1\Sigma_g^+$ to a $^3\Sigma_g^+$	
		12.3-14.7	Electron excitation from X $^1\Sigma_g^+$ to C $^1\Pi_u$	
		12.3-14.4	Electron excitation from X $^1\Sigma_g^+$ to E(F) $^1\Sigma_g^+$	
CO_2	11.2-11.7	11.0-11.8	Electron excitation from X $^1\Sigma_g^+$ to $^1\Sigma_u^+$	1. *J. Chem. Phys.* 2003, 119, 9628-9632; 2. *J. Phys. B* 1988, 21, 3211; 3. *J. Chem. Phys.* 1984, 80, 648-656; 4. J. Chem. Phys. 1979, 70, 1711-1719; 5. *Chem. Phys. Lett.* 1973, 20, 489.
		11.2	Electron excitation from X $^1\Sigma_g^+$ to $^3\Sigma_u^-$	
		11.4	Electron excitation from X $^1\Sigma_g^+$ to $^1\Pi_g$	
		11.4	Electron excitation from X $^1\Sigma_g^+$ to $^{3,1}\Pi_u$	
	13.1-13.5	12.6-13.8	First ionization transition from X $^1\Sigma_g^+$ to CO_2^+ $^2\Pi_g$	
	15.8-16.0	15.5-18.1	Ionization transition from X $^1\Sigma_g^+$ to CO_2^+ $^2\Sigma_u^+$	
	17.7-18.2			
N_2	12.9-13.1	12.7-14.3	Electron excitation from X $^1\Sigma_g^+$ to $^2\Pi_g$	1. *J. Chem. Phys.* 2002, 117, 4348–4360; 2. *J. Chem. Phys.* 2006, 124, 124311; 3. *J. Phys. B: At. Mol. Opt. Phys.* 1996, 29, 5389; 4. *J. Phys. B: Atom. Mol. Opt. Phys.* 1992, 25, 135.
	14.0-14.1			
	15.9-16.0	16.3-18.7	Electron excitation from X $^1\Sigma_g^+$ to $^2\Sigma_g^+$	
	17.6-18.1			

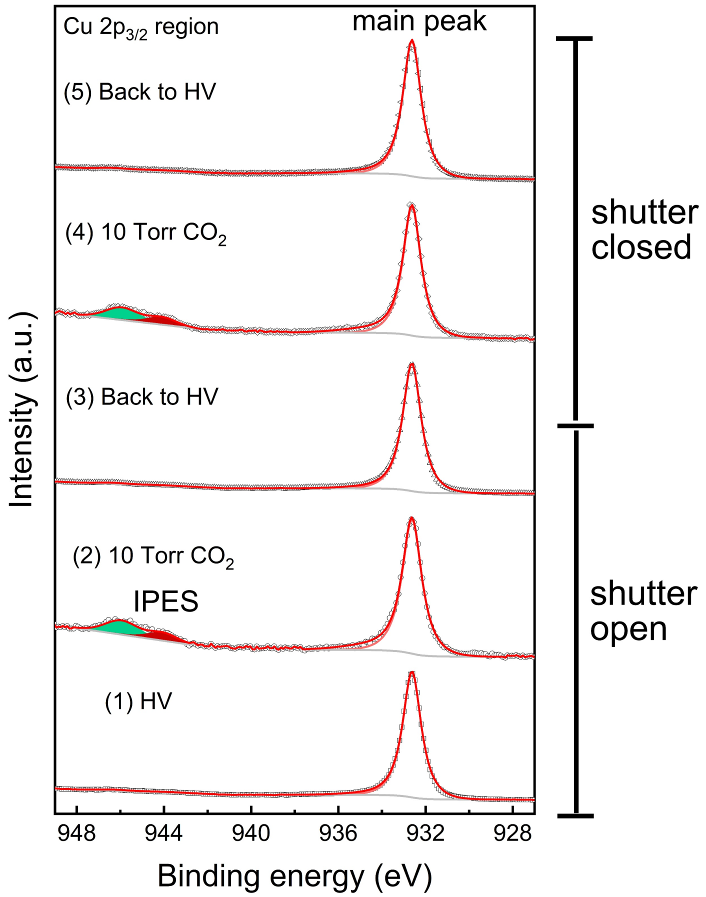

Figure S2. Cu $2p_{3/2}$ photoelectron peaks and deconvolution results (photon energy = 4 keV) on the surface of the polycrystalline Cu metal foil at the sequent conditions of (1) HV, (2) 10 Torr CO_2, (3) Back to HV, (4) 10 Torr CO_2, and (5) back to HV, respectively.

Beam-induced damage via radiolysis is a common occurrence in X-ray core-level spectroscopies, stemming from ionic fragmentation and interactions of secondary electrons during photoionization and relaxation processes. Increasing gas pressure within a chamber volume heightens the likelihood of scattering, thereby augmenting energy absorption and radical production from gas molecules. Excitation with soft X-rays yields larger photoionization cross-sections of core-level electrons. Due to the relatively low kinetic energies and mean-free paths of photoelectrons from soft X-rays, XPS exhibits nanometer-scale interface sensitivity. However, significant energy loss occurs near the irradiated interface. Therefore, in ambient-pressure X-ray spectroscopy, careful consideration of beam-induced damage is imperative when examining interfacial behaviors. This is due to the interaction of reactive species generated by radiolysis with the metal surface. Studies have shown that the oxidation states of Cu metal surfaces are substantially influenced by soft X-rays, leading to the oxidation of Cu metal to CuO[3-6].

Here, to eliminate the possibility of beam-induced Cu oxidation, we conducted the following

control experiments and obtained Figure S2. Firstly, we collected the high-resolution Cu 2p spectrum under high vacuum (HV) conditions, where photoelectron scattering features were not observed. Next, with the shutter open to maintain continuous X-ray illumination on the Cu metal foil, we introduced CO_2 into the main chamber of the AP-XPS end-station to reach a pressure of 10 Torr (condition 2). The sample was equilibrated, and time-dependent scans of the Cu 2p region were conducted until stable spectra were obtained (this process took approximately 10 minutes). Subsequently, high-resolution measurements of the Cu 2p spectrum were performed, revealing the presence of photoelectron scattering features. Then, while keeping the shutter open, we removed CO_2 from the main chamber to return to the HV condition (condition 3). During this process, the sample was equilibrated, and time-dependent scans of the Cu 2p region were recorded. After obtaining stable spectra, high-resolution Cu 2p spectrum measurements were taken, and the scattering satellite peaks vanished. In the subsequent step, with the shutter closed to cease X-ray illumination on the sample, CO_2 gas was introduced into the main chamber to reach a pressure of 10 Torr (condition 4). After equilibrating the sample for 10 minutes under this condition, the shutter was opened, and high-resolution Cu 2p spectrum measurements were conducted. The photoelectron satellite peaks were observed again, confirming that the scattering satellite signatures were induced solely by CO_2. Following this, the shutter was promptly closed, and CO_2 gas was evacuated from the main chamber to return to the HV condition (condition 5). After equilibrating the sample for 10 minutes under these conditions, the shutter was reopened, and high-resolution Cu 2p spectrum measurements were recorded. The shake-up satellite peaks disappeared, thus concluding that beam-induced damage did not contribute to the observed photoelectron scattering features.

Table S2. The standard deviations of I_s/I_m data points in **Figure 3c** calculated by the corresponding standard deviations of I_s and I_m from XPS spectra fitting.

Pressure (Torr)	Standard deviation
0.001	0.000375198
0.5	0.000296003
0.75	0.00028774
1	0.00029588
2	0.000357074
4	0.000442339
6	0.000706889
8	0.000930846
10	0.001233473
12.5	0.001461168
15	0.001663111
20	0.003601495

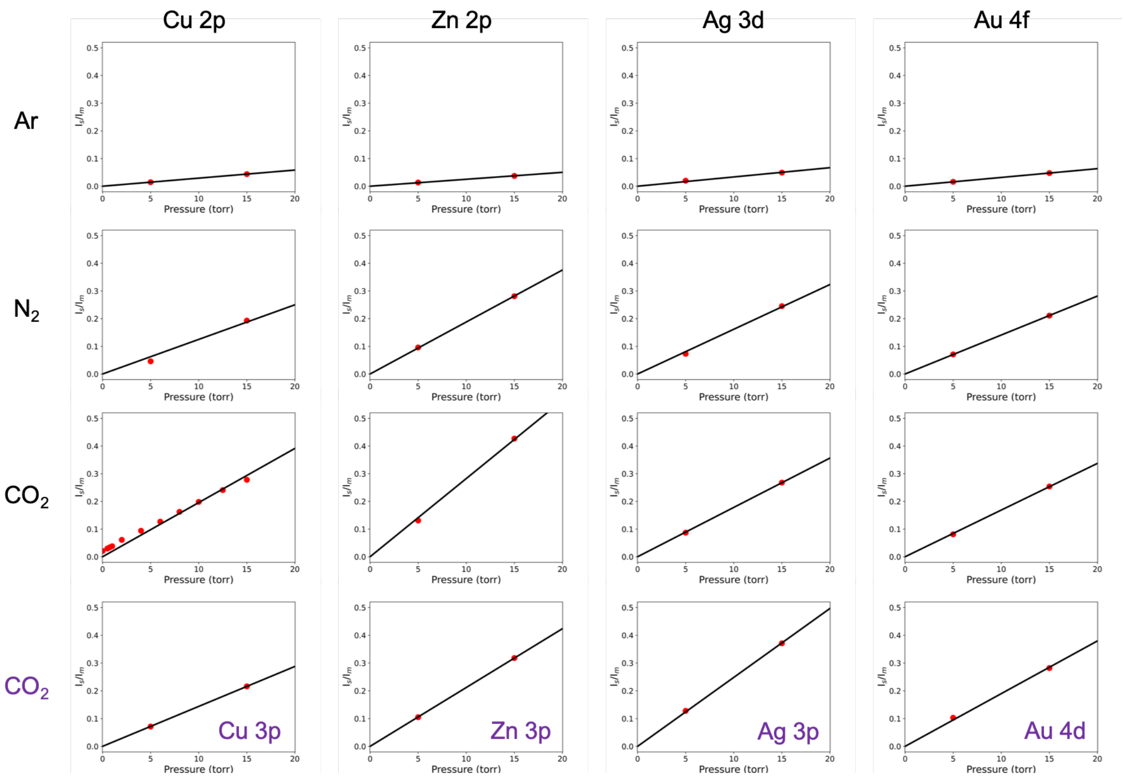

Figure S3. The ratio of scattering to main peak intensity (I_s/I_m) as a function of pressure for the first few gas transitions in every metal/gas pair from corresponding XPS spectra. Without gas molecules (pressure = 0), there is no IPES induced by gas molecules and thus $I_s/I_m = 0$. Thus, the curves start from the point, (0, 0), and intensity ratio is linearly proportional to the pressure. The experimental data points are represented by the red solid dots.

Table S3. The standard deviations of I_s/I_m data points in **Figure S3** calculated by the corresponding standard deviations of I_s and I_m from XPS spectra fitting. The standard deviations of I_s/I_m data points from Cu 2p region in the CO_2 environment in **Figure S3** are exhibited in **Table S2**.

Gas	Ar		N_2		CO_2	
Pressure (Torr)	5	15	5	15	5	15
Au 4f	0.00014116	0.000271	0.00032	0.000654	0.000441	0.001095
Ag 3d	0.00012349	0.000237	0.000267	0.000598	0.000451	0.00101
Zn 2p	7.208E-05	0.000145	0.000176	0.000396	0.000277	0.001673
Cu 2p	6.4467E-05	0.000166	9.6E-05	0.00021	N/A	N/A
Au 4d					0.000601	0.001706
Ag 3p					0.001499	0.004486
Zn 3p					0.000452	0.002204
Cu 3p					0.000604	0.001593

Table S4. The slopes obtained by linearly fitting intensity ratios of IPES peak to the main peak and the gas pressure for metal /gas pairs.

Gas	CO_2			N_2			Ar		
Region	KE (eV)	Slope ($Torr^{-1}$)	R^2	KE (eV)	Slope ($Torr^{-1}$)	R^2	KE (eV)	Slope ($Torr^{-1}$)	R^2
Au 4f	3916	0.019	0.995	3916	0.014	1	3916	0.003	1
Ag 3d	3632	0.018	1	3632	0.016	0.995	3632	0.003	0.972
Zn 2p	2978	0.028	0.997	2978	0.019	1	2978	0.003	0.996
Cu 2p	3067	0.02	0.968	3067	0.013	0.972	3067	0.003	1
Au 4d	3665	0.017	0.999						
Ag 3p	3427	0.025	0.999						
Zn 3p	3911	0.021	1						
Cu 3p	3923	0.014	1						

Table S5. Comparison of averaged electron excitation cross section (σ) at the photoelectron kinetic energy range of 2800-4000 eV between this work and other reports.

Gas	σ (CO_2) / cm^2	σ (N_2) / cm^2	σ (Ar) / cm^2
This work	$1.79 \times 10^{-17} \pm 3.68 \times 10^{-18}$	$1.37 \times 10^{-17} \pm 2.02 \times 10^{-18}$	$2.3 \times 10^{-18} \pm 0$
Other reports	1.7×10^{-17}	1.0×10^{-17}	5.6×10^{-18}
Reference	7	8	9

REFERENCES

(1) Li, R.; Cheng, W.-H.; Richter, M. H.; DuChene, J. S.; Tian, W.; Li, C.; Atwater, H. A. Unassisted Highly Selective Gas-Phase CO_2 Reduction with a Plasmonic Au/p-GaN Photocatalyst Using H_2O as an Electron Donor. *ACS Energy Lett.* **2021,** *6* (5), 1849-1856.

(2) Prabhakar, R. R.; Lemerle, R.; Barecka, M.; Kim, M.; Seo, S.; Dayi, E. N.; Dei Tos, I.; Ager, J. W. TaO_x Electron Transport Layers for CO_2 Reduction Si Photocathodes. *J. Mater. Chem. A* **2023,** *11* (25), 13588-13599.

(3) Feijóo, J.; Yang, Y.; Fonseca Guzman, M. V.; Vargas, A.; Chen, C.; Pollock, C. J.; Yang, P. *Operando* High-Energy-Resolution X-ray Spectroscopy of Evolving Cu Nanoparticle Electrocatalysts for CO_2 Reduction. *J. Am. Chem. Soc.* **2023,** *145* (37), 20208-20213.

(4) Weatherup, R. S.; Wu, C. H.; Escudero, C.; Pérez-Dieste, V.; Salmeron, M. B. Environment-Dependent Radiation Damage in Atmospheric Pressure X-ray Spectroscopy. *J. Phys. Chem. B* **2018,** *122* (2), 737-744.

(5) Yang, Y.; Louisia, S.; Yu, S.; Jin, J.; Roh, I.; Chen, C.; Fonseca Guzman, M. V.; Feijóo, J.; Chen, P.-C.; Wang, H.; Pollock, C. J.; Huang, X.; Shao, Y.-T.; Wang, C.; Muller, D. A.; Abruña, H. D.; Yang, P. *Operando* studies reveal active Cu nanograins for CO_2 electroreduction. *Nature* **2023,** *614* (7947), 262-269.

(6) Yang, Y.; Roh, I.; Louisia, S.; Chen, C.; Jin, J.; Yu, S.; Salmeron, M. B.; Wang, C.; Yang, P. *Operando* Resonant Soft X-ray Scattering Studies of Chemical Environment and Interparticle Dynamics of Cu Nanocatalysts for CO_2 Electroreduction. *J. Am. Chem. Soc.* **2022,** *144* (20), 8927-8931.

(7) Lozano, A. I.; García-Abenza, A.; Blanco Ramos, F.; Hasan, M.; Slaughter, D. S.; Weber, T.; McEachran, R. P.; White, R. D.; Brunger, M. J.; Limão-Vieira, P.; García Gómez-Tejedor, G. Electron and Positron Scattering Cross Sections from CO_2: A Comparative Study over a Broad Energy Range (0.1–5000 eV). *J. Phys. Chem. A* **2022,** *126* (36), 6032-6046.

(8) Song, M.-Y.; Cho, H.; Karwasz, G. P.; Kokoouline, V.; Tennyson, J. Cross Sections for Electron Collisions with N_2, N_2^*, and N_2^+. *J. Phys. Chem. Ref. Data* **2023,** *52* (2), 023104.

(9) Heer, F. J. d.; Jansen, R. H. J.; Kaay, W. v. d. Total cross sections for electron scattering by Ne, Ar, Kr and Xe. *J. Phys. B: Atom. Mol. Phys.* **1979,** *12* (6), 979.
