# Supplementary material for: When Photoelectrons Meet Gas Molecules: Determining the Role of Inelastic Scattering in Ambient Pressure X-ray Photoelectron Spectroscopy
Source: ACS Cent Sci. 2024 Dec 20;11(1):98–106. doi: 10.1021/acscentsci.4c01841 (PMC11758507; doi:10.1021/acscentsci.4c01841)
Supplement: Supplementary file 2 — oc4c01841_si_002.pdf [file oc4c01841_si_002.pdf]

oc-2024-01841n.R1

Name: Peer Review Information for "When Photoelectrons Meet Gas Molecules: Determining the Role of Inelastic Scattering in Ambient Pressure X-ray Photoelectron Spectroscopy"

First Round of Reviewer Comments

Reviewer: 1

Comments to the Author

Review oc-2024-01841n

by Li and co-workers

The manuscript deals with a relevant topic related to core-level XPS spectra on different metal surfaces showing the universal nature of IPES across different systems. Moreover, IPES is here shown to be independent of the composition, structure, or size of the solid materials.

Authors have comprehensively proved the universality and impacts of IPES in the APXPS system, by investigating two mechanisms on CO2 reduction systems (p-GaN/Au/Cu and p-Si/TaOx/Cu), thus showing that IPES structures are independent of the composition, structure, and size of the solid materials. This is a relevant achievement.

From the IPES structures identified, the authors' analytical method seems to accurately describe the correlation between the intensity ratio of the IPES to the main peaks, allowing for the determination of the electronic excitation cross sections of the targeted gases. As a consequence, they are able to obtain the electronic excitation cross sections of gas molecules.

I am convinced of the relevance of this new approach and description, and before the manuscript can be accepted, I would like authors to address a couple of issues which may need clarified, since the outcome of this research may attract the interest of a larger research community that may in some particular cases not be too familiar with a few details. Thus, any comment and/or adding a short sentence to the manuscript main body or the SI will be important.

Figures 1 and 2, the fitting usually does not fully reproduce the main peaks experimental data left branches mainly under HV conditions, in Figure 1 more evident in Au 4f and Ag 3d, whereas in Figure 2 in d)-h).

As far as Figure 3 is concerned, we note the underlying contribution to the left of the main peak more "relevant" for p-GaN/Au/Cu. Is this related to a certain extent to the Au nanoparticles? please clarify.

Figure 4, apart from Ar, and within the uncertainty plotted, there is no significant difference from previous work. If such is expected, then  suggest a short sentence to be added to address this.

Reviewer: 2

Comments to the Author

This paper described experiments and analysis of inelastic photoelectron scattering (IPES) peaks which are observed in ambient pressure X-ray photoelectron spectroscopy (APXPS). APXPS spectra were recorded in a number of spectral regions at high vacuum conditions and in the presence of low pressures of H2, CO2, N2 and Ar. Clear IPES features observed in these spectra were characterize, both with and without the presence of polycrystalline metal foils. Spectra were also measured in the presence of several gas mixtures. The measurements enabled the unambiguous identification and assignment of the IPES features observed. Studies of the IPES peak intensities as a function of pressure enabled the determination of inelastic scattering cross sections which were found to agree reasonably well with literature values.

The paper presents new results which should be of interest of users of APXPS. The papers is clear, and the figures well-illustrated. The material described represents a solid piece of experimental and modelling work: it will undoubtedly be of interest to the APXPS community, but I do not find the material covered particularly novel or surprising. Nevertheless, on ballance, I believe the paper is probably acceptable for publication in ACS Central Science.

I have a couple of minor points for the authors to address.

1. Many of the IPES peaks shown in figures 1 and 2 are broad and overlapping. In the figures the individual peaks are highlighted with different coloured profiles, but it was unclear how there were obtained. In many cases the separate peaks are not well resolved, so I believe there must be an element of ambiguity in some of these assignments.

2. One of the more interesting questions in my mind is the nature of the excitations responsible for the IPES peaks. There is a brief comment of the assignments on p5 of the manuscript, which is not that easy to follow due to some typographical issues. I felt the assignments deserved a more careful explanation.

3. There appears to be a problem with the text in some parts of the supplementary - perhaps this is a corrupted pdf file that needs fixing before publication.

Author's Response to Peer Review Comments:

November 29, 2024

Editors of *ACS Central Science*

Dear Editor,

We sincerely thank you for considering our manuscript, titled "When Photoelectrons Meet Gas Molecules: Determining the Role of Inelastic Scattering in Ambient Pressure X-ray Photoelectron Spectroscopy" (Manuscript ID: oc-2024-01841n) for publication in *ACS Central Science*. We are grateful for the insightful comments and valuable suggestions from you and the Reviewers, which have been helpful in enhancing the quality of our manuscript. Based on these recommendations, we have made several revisions to the original manuscript, with the main changes (in red) in the revised manuscript. Our point-by-point responses to the reviewers' comments (in blue) are provided in the following pages. The key revisions include:

1. Revised Figure 1, 2, 3 and S1 in response to Reviewers' feedback.
2. Added further discussion and new references in the revised manuscript to address specific Reviewers' comments.
3. Added Table S1 to the revised Supporting Information in response to the Reviewer's comments.
4. Corrected the display issues in the legends of Tables S2–S4 in the revised Supporting Information.
5. Improved the language and revised the typographical and formatting errors in revised manuscript and Supporting Information.
6. Modified the ToC figure and added a synopsis to meet the standard of the journal.

We hope this revised version meets the high standards of *ACS Central Science*. Your

consideration of our manuscript for acceptance and publication would be greatly appreciated. Finally, all authors have reviewed the revised manuscript and have approved this submission. We look forward to your response.

Sincerely,

Junko Yano, PhD
*Liquid Sunlight Alliance*
*Molecular Biophysics & Integrated Bioimaging Division*
*Lawrence Berkeley National Laboratory*

Ethan J. Crumlin, PhD
*Chemical Sciences Division*
*Advanced Light Source*
*Lawrence Berkeley National Laboratory*

**Reviewer: 1**

Recommendation: Publish in ACS Central Science after minor revisions noted.

Comments:
Review oc-2024-01841n
by Li and co-workers

The manuscript deals with a relevant topic related to core-level XPS spectra on different metal surfaces showing the universal nature of IPES across different systems. Moreover, IPES is here shown to be independent of the composition, structure, or size of the solid materials.

Authors have comprehensively proved the universality and impacts of IPES in the APXPS system, by investigating two mechanisms on CO2 reduction systems (p-GaN/Au/Cu and p-Si/TaOx/Cu), thus showing that IPES structures are independent of the composition, structure, and size of the solid materials. This is a relevant achievement.

From the IPES structures identified, the authors' analytical method seems to accurately describe the correlation between the intensity ratio of the IPES to the main peaks, allowing for the determination of the electronic excitation cross sections of the targeted gases. As a consequence, they are able to obtain the electronic excitation cross sections of gas molecules.

I am convinced of the relevance of this new approach and description, and before the manuscript can be accepted, I would like authors to address a couple of issues which may need clarified, since the outcome of this research may attract the interest of a larger research community that may in some particular cases not be too familiar with a few details. Thus, any comment and/or adding a short sentence to the manuscript main body or the SI will be important.

**Response:** We greatly appreciate the positive response by the Reviewer. The detailed comments raised by the Reviewer have been addressed point-by-point below. With this, we hope the revised manuscript is now suitable for publication in *ACS Central Science*.

1. Figures 1 and 2, the fitting usually does not fully reproduce the main peaks experimental data left branches mainly under HV conditions, in Figure 1 more evident in Au 4f and Ag 3d, whereas in Figure 2 in d)-h).

**Response:** Thank you for your observation regarding the fitting discrepancies in **Figures 1** and **2**, particularly for the left branches of the main peaks under high vacuum (HV) conditions. We appreciate the opportunity to address this point and improve the clarity of our spectra presentation.

To address your concern, we have revisited the fitting procedure and further optimized the

fitting parameters to better align the fitting curves with the raw data, particularly for the Au 4f and Ag 3d spectra in **Figure 1** and **Figure 2 (d)-(h)**. These adjustments involved refining the baseline subtraction, peak asymmetry modeling, and overall fit quality. The updated fitting results show an improved match with the raw data, while maintaining the integrity and reliability of the peak analysis. We hope these refinements address your concern while adhering to the widely accepted practices in XPS data processing.

**Revisions made**: We have revised **Figure 1**, **2** and **S1** in the revised manuscript and Supporting Information as follows.

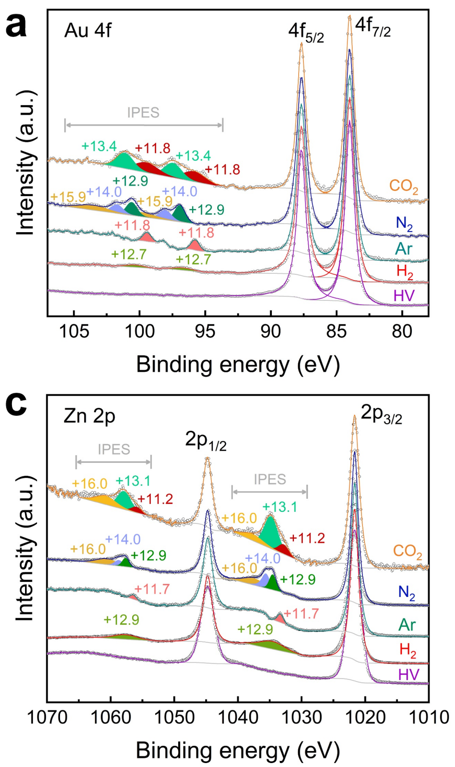


**Figure 1.** IPES features induced by the surrounding gas molecules in the core-level spectra of metal solids. Deconvoluted IPES structures (solid-colored areas) are identified by the differences in BE compared to that of the main peaks in the (a) Au 4f, (b) Ag 3d, (c) Zn 2p and (d) Cu 2p regions collected at room temperature under HV; 5 Torr of $H_2$; and 15 Torr of $CO_2$, $N_2$, and Ar conditions. The differences in BEs between each pair of satellite peak(s) and main lines are indicated above the corresponding IPES structures.

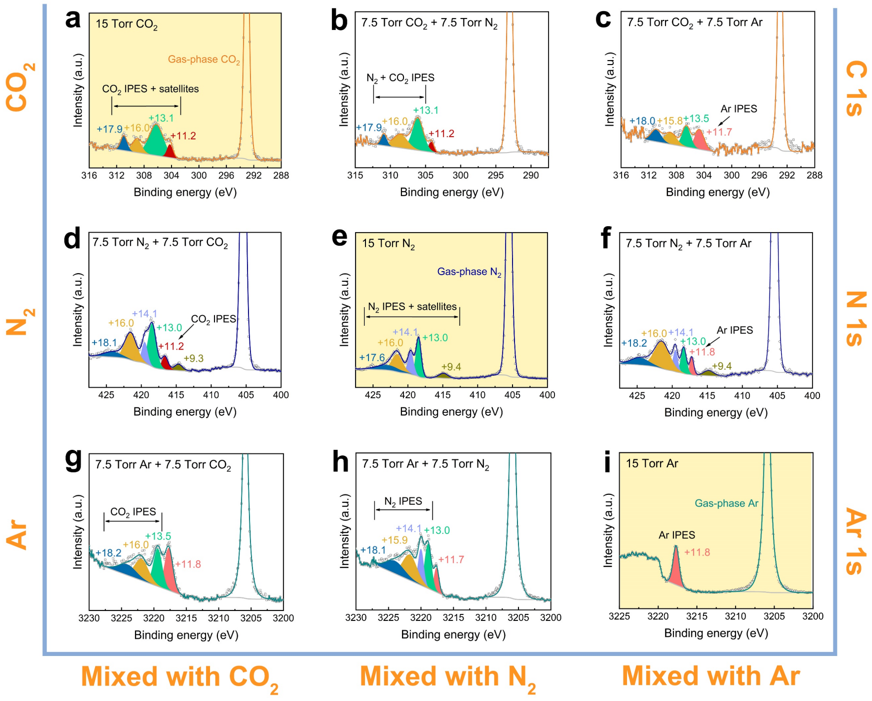


**Figure 2.** Gas-phase XPS spectra without metal solids, illustrating the gas-dependent interactions between photoelectrons and gas molecules. Deconvoluted IPES structures in the (a), (b), (c) C 1s, (d), (e), (f) N 1s, and (g), (h), (i) Ar 1s regions are identified by differences in BE from the main peaks, collected under pure gases and pairwise mixtures of $CO_2$, $N_2$, and Ar at a total pressure of 15 Torr. The yellow background highlights the spectra collected under single-gas conditions, distinguishing them from those collected under pairwise-gas conditions.

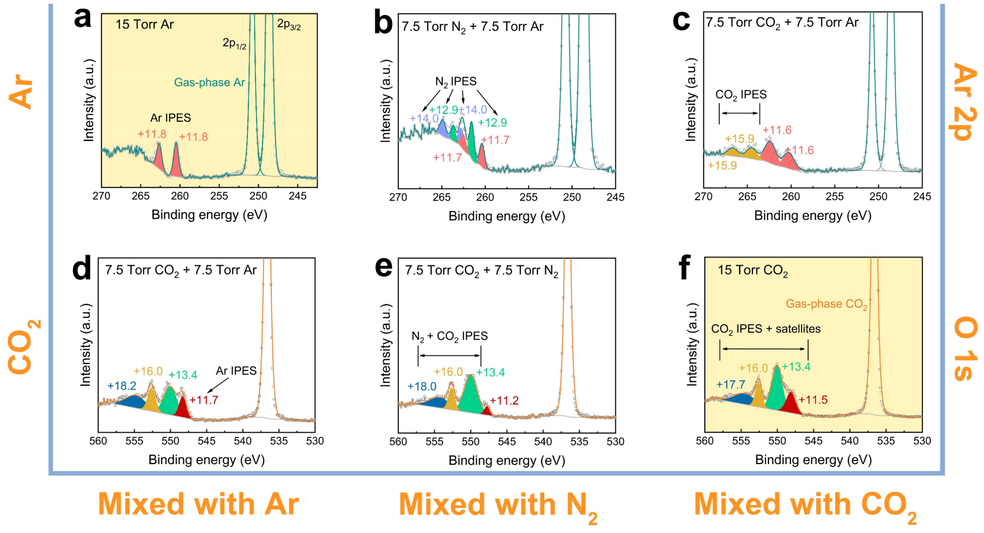


**Figure S1**. Gas-phase XPS spectra without metal foil samples illustrate gas-dependent interaction between photoelectrons and gas molecules. Deconvoluted scattering signatures in

(a), (b), (c) Ar 2p and (d), (e), (f) O 1s regions are identified by the differences in binding energy from the main peaks collected under pure and pairwise mixtures of Ar, $N_2$, and $CO_2$ at the total pressure of 15 Torr, respectively. The yellow background highlights the spectra collected under single-gas conditions, distinguishing them from those collected under pairwise-gas conditions.

2. As far as Figure 3 is concerned, we note the underlying contribution to the left of the main peak more "relevant" for p-GaN/Au/Cu. Is this related to a certain extent to the Au nanoparticles? please clarify.

**Response:** Thank you for pointing out the underlying contribution to the left of the main peak in the Cu 2p region for p-GaN/Au/Cu in **Figure 3**. The observed underlying peak corresponds to $Cu^{2+}$, which is unrelated to the presence of Au nanoparticles. This Cu feature arises from the oxidation of Cu nanoparticles due to air exposure. The Cu nanoparticles in our system have a size of approximately 5 nm, making them particularly susceptible to oxidation upon air exposure, as compared to Cu thin films (e.g., p-Si/$TaO_x$/Cu) or bulk Cu metal foil. Additionally, being nanoparticles, they have a larger surface to bulk ratio than thin films or bulk foils thus resulting in a larger surface oxidation to bulk metal ratio. Based on prior studies (*ACS Energy Lett.* 2021, 6, 1849-1856; *ACS Nano* 2024, 18, 19538-19548.), the Cu species could include CuO and $CuCO_3 \cdot Cu(OH)_2$ (malachite), both of which are commonly formed on the surfaces of Cu nanoparticles upon exposure to ambient conditions (*Appl. Surf. Sci.* 2019, 473, 25-30; *Electrochim. Acta* 2013, 111, 771-778.). This has been corroborated by XPS and X-ray absorption near-edge spectroscopy (XANES) spectra in previous works using the same catalyst system, which also identified the presence of $Cu^{2+}$ on the as-prepared p-GaN/Au/Cu sample (*ACS Energy Lett.* 2021, 6, 1849-1856; *ACS Nano* 2024, 18, 19538-19548.). To further verify this, we also collected XPS spectra for the Cu 2p region (Figure R1) on a control sample (p-GaN/Cu) where Cu nanoparticles were directly deposited onto the p-GaN surface without Au nanoparticles. The spectrum similarly revealed a $Cu^{2+}$ peak at the similar binding energy, confirming that the oxidation of Cu nanoparticles is independent of the Au nanoparticles and is instead due to their inherent susceptibility to oxidation in air.

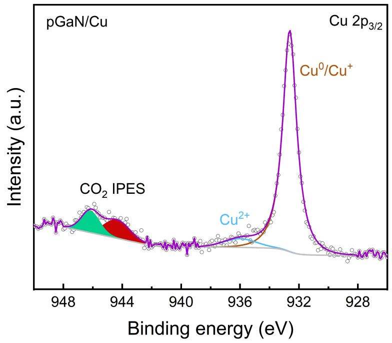


**Figure R1.** Deconvoluted Cu $2p_{3/2}$ region of XPS spectrum collected under 15 Torr of $CO_2$ on p-GaN/Cu.

**Revisions made**: (1) We have added the following discussion to the revised manuscript.

"As shown in **Figure 3a**, the p-GaN/Au/Cu system features small Cu nanoparticles (5 nm) supported by larger Au nanoparticles (40 nm) on a p-GaN substrate. The Cu nanoparticles contained a small amount of $Cu^{2+}$, likely resulting from air exposure[19]."

(2) We have revised **Figure 3a** in the revised manuscript as follows.

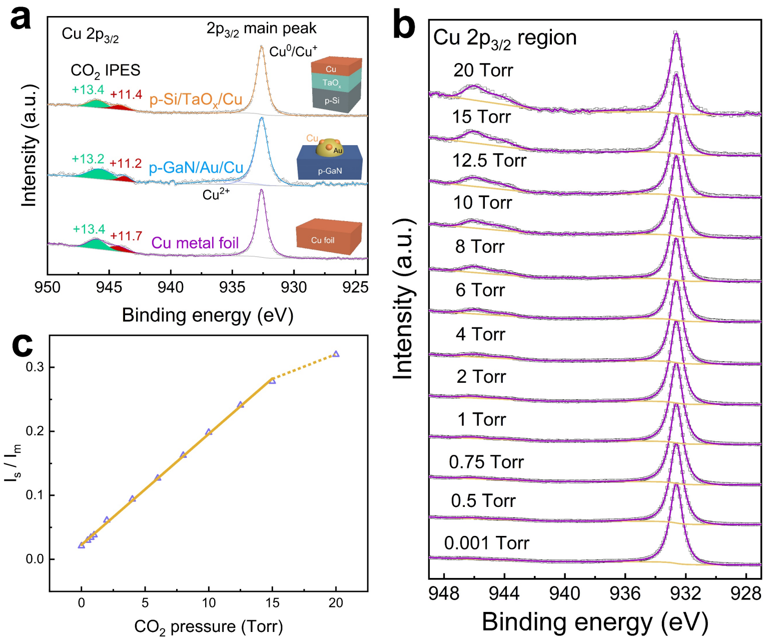


**Figure 3.** The independence of IPES on the composition, morphology, and size of the solid materials, as well as pressure-dependent IPES features induced by gas molecules. (a) Deconvoluted Cu $2p_{3/2}$ regions collected under 15 Torr of $CO_2$ on p-GaN/Au/Cu, p-Si/$TaO_x$/Cu and polycrystalline Cu metal foil. The inset schemes show the structure and composition of the three materials systems. (b) Cu $2p_{3/2}$ spectra collected on the Cu metal foil with increasing $CO_2$ pressure. (c) Calculated area ratio between IPES and main peaks ($I_s/I_m$) as a function of $CO_2$ pressure. The linear fitting curve was obtained between 0.001 and 15 Torr of $CO_2$. The corresponding slope and intercept are $0.017 \pm 1.57\times10^{-4}$ $Torr^{-1}$ and $0.023 \pm 1.16\times10^{-3}$, respectively, and the coefficient of determination is 0.999.

3. Figure 4, apart from Ar, and within the uncertainty plotted, there is no significant difference from previous work. If such is expected, then suggest a short sentence to be added to address this.

**Response:** We thank the Reviewer for highlighting this point and providing us with the opportunity to further clarify it. While the electronic excitation cross section values for $N_2$ and $CO_2$ align well with those in the existing references, the value for Ar shows a noticeable

discrepancy. This difference arises because our calculated electronic excitation cross section corresponds to the first few electronic transitions in the gases, whereas the literature values represent the sum of all possible electronic transitions. Since the first few electronic transitions typically have the highest probabilities and dominate the scattering peak intensities, we approximated the electronic excitation cross sections using these partial values. However, it is more accurate to state that the electronic excitation cross section values reported in the literature, which include all transitions, are expected to be larger and serve as an upper bound for our results. This distinction is evident in the case of Ar. For $N_2$ and $CO_2$, the literature values were estimated by published figures, which might introduce some uncertainties (*J. Phys. Chem. Ref. Data* 2023, 52, 023104; *J. Phys. Chem. A* 2022, 126, 6032-6046). Specifically, for $N_2$, the reported value represents a lower bound. Despite these differences, we used the literature values as a benchmark to ensure that the order of magnitude of our calculated electronic excitation cross sections is reasonable. In this work, we mainly aim to introduce a novel methodology to obtain the electronic excitation cross sections of gas molecules, providing valuable insights for the APXPS community.

**Revisions made**: We have added the following discussion to the revised manuscript.
"Based on the slopes of other gas/metal pairs (Figure S3 and Table S4), we calculated the average electronic excitation cross sections (σ) for Ar, $N_2$, and $CO_2$, which are comparable with the values obtained using other experimental techniques for photoelectron KEs between 2800 and 4000 eV (Figure 4 and Table S5). While the electronic excitation cross sections for $N_2$ and $CO_2$ align well with those in the existing references, the value for Ar shows a minor discrepancy. This difference arises because our calculated electronic excitation cross section corresponds to the first few electronic transitions in the gases, whereas the values in the references represent the sum of all possible electronic transitions. Despite these differences, we used the literature values as a benchmark to ensure that the order of magnitude of our calculated electronic excitation cross sections is reasonable."

**Thanks again for the valuable comments and suggestions from the reviewer. We have carefully addressed each point to meet the high standards expected.**

**Reviewer: 2**

Recommendation: Publish in ACS Central Science after minor revisions noted.

Comments:

This paper described experiments and analysis of inelastic photoelectron scattering (IPES) peaks which are observed in ambient pressure X-ray photoelectron spectroscopy (APXPS). APXPS spectra were recorded in a number of spectral regions at high vacuum conditions and in the presence of low pressures of H2, CO2, N2 and Ar. Clear IPES features observed in these spectra were characterize, both with and without the presence of polycrystalline metal foils. Spectra were also measured in the presence of several gas mixtures. The measurements enabled the unambiguous identification and assignment of the IPES features observed. Studies of the IPES peak intensities as a function of pressure enabled the determination of inelastic scattering cross sections which were found to agree reasonably well with literature values.

The paper presents new results which should be of interest of users of APXPS. The papers is clear, and the figures well-illustrated. The material described represents a solid piece of experimental and modelling work: it will undoubtedly be of interest to the APXPS community, but I do not find the material covered particularly novel or surprising. Nevertheless, on ballance, I believe the paper is probably acceptable for publication in ACS Central Science.

I have a couple of minor points for the authors to address.

**Response:** We thank the Reviewer for the praise of this work. The detailed point-by-point responses have been shown below. Based on this, we hope the revised manuscript is now suitable for publication in *ACS Central Science*.

1. Many of the IPES peaks shown in figures 1 and 2 are broad and overlapping. In the figures the individual peaks are highlighted with different coloured profiles, but it was unclear how there were obtained. In many cases the separate peaks are not well resolved, so I believe there must be an element of ambiguity in some of these assignments.

**Response:** We appreciate the Reviewer's insightful comment, which allows us to further clarify the methodology used to analyze the IPES structures and address the observed ambiguities in peak assignments. The individual IPES structures highlighted in **Figures 1** and **2** by solid colors were determined based on the BE values reported in the literature, which were regarded as the XPS curve-fitting constraints. This approach ensures that our assignments are guided by established reference results. We recognize that some peaks in the experimental spectra are broad and overlapping, which can introduce challenges in fully resolving individual features. However, the use of BEs reported in literature as benchmarks provides a reliable foundation for identifying and deconvoluting these IPES structures.

In the revised Supporting Information, we have included Table R1 as Table S1 that compares the BE values obtained from our experiments with those reported in the literature. This comparison will demonstrate the consistency of our assignments and provide transparency regarding the alignments between our results and established references.

**Table R1.** The comparison of the BE values obtained from our experiments with those reported in the references. Peak assignments were exhibited as the possible excitation states shown in the references, especially those in the molecular orbitals of $H_2$, $CO_2$ and $N_2$. Broad fitting peaks were used in the metal and gas-phase core-level spectra to fit the IPES structures in **Figure 1**, **2** and **S1**, which may account for multiple transitions that occur with closely spaced transition energies, as shown in the electron energy loss spectroscopy (EELS) for the gases in the references.

| Gas | Our BEs (eV) | Literature BEs (eV) | Peak Assignment | References |
|---|---|---|---|---|
| Ar | 11.6-11.8 | 11.6-11.8 | Electron transition from the $3p^6$ to $3p^5 4s^1$ orbital | 1. *J. Electron Spectrosc.* 2019, 232, 111-120; 2. *J. Chem. Phys.* 1968, 48, 5066-5096. |
| $H_2$ | 12.7-12.9 | 11.2-14.6 | Electron excitation from X $^1\Sigma_g^+$ to B $^1\Sigma_g^+$ | 1. *J. Phys. B: At. Mol. Opt. Phys.* 2002, 35, 4695; 2. *J. Phys. B: At. Mol. Opt. Phys.* 2017, 50, 225203; 3. *J. Mol. Spectrosc.* 1969, 32, 39-53; 4. *J. Chem. Phys.* 1966, 44, 440-441; 5. *J. Mol. Spectrosc.* 1972, 41, 425-486. |
| | | 11.8-14.7 | Electron excitation from X $^1\Sigma_g^+$ to c $^3\Pi_u$ | |
| | | 11.8-14.7 | Electron excitation from X $^1\Sigma_g^+$ to a $^3\Sigma_g^+$ | |
| | | 12.3-14.7 | Electron excitation from X $^1\Sigma_g^+$ to C $^1\Pi_u$ | |
| | | 12.3-14.4 | Electron excitation from X $^1\Sigma_g^+$ to E(F) $^1\Sigma_g^+$ | |
| $CO_2$ | 11.2-11.7 | 11.0-11.8 | Electron excitation from X $^1\Sigma_g^+$ to $^1\Sigma_u^+$ | 1. *J. Chem. Phys.* 2003, 119, 9628-9632; 2. *J. Phys. B* 1988, 21, 3211; 3. *J. Chem. Phys.* 1984, 80, 648-656; 4. J. Chem. Phys. 1979, 70, 1711-1719; 5. *Chem. Phys. Lett.* 1973, 20, 489. |
| | | 11.2 | Electron excitation from X $^1\Sigma_g^+$ to $^3\Sigma_u^-$ | |
| | | 11.4 | Electron excitation from X $^1\Sigma_g^+$ to $^1\Pi_g$ | |
| | | 11.4 | Electron excitation from X $^1\Sigma_g^+$ to $^{3,1}\Pi_u$ | |
| | 13.1-13.5 | 12.6-13.8 | First ionization transition from X $^1\Sigma_g^+$ to $CO_2^+$ $^2\Pi_g$ | |
| | 15.8-16.0 | 15.5-18.1 | Ionization transition from X $^1\Sigma_g^+$ to $CO_2^+$ $^2\Sigma_u^+$ | |
| | 17.7-18.2 | | | |
| $N_2$ | 12.9-13.1 | 12.7-14.3 | Electron excitation from X $^1\Sigma_g^+$ to $^2\Pi_g$ | 1. *J. Chem. Phys.* 2002, 117, 4348–4360; 2. *J. Chem. Phys.* 2006, 124, 124311; 3. *J. Phys. B: At. Mol. Opt. Phys.* 1996, 29, 5389; 4. *J. Phys. B: Atom. Mol. Opt. Phys.* 1992, 25, 135. |
| | 14.0-14.1 | | | |
| | 15.9-16.0 | 16.3-18.7 | Electron excitation from X $^1\Sigma_g^+$ to $^2\Sigma_g^+$ | |
| | 17.6-18.1 | | | |

Furthermore, as we discussed in the manuscript, the observed shifts of less than 0.6 eV to higher BEs compared to theoretical values can be attributed to two primary factors (*J. Electron Spectrosc.* 2019, 232, 111-120; *Appl. Surf. Sci.* 2020, 530, 147243.):

1. Experimental Broadening: The larger full width at half maximum (FWHM) of the IPES structures in our spectra is influenced by the high gas pressures used in our experiments. This leads to peak broadening and overlapping caused by additional scattering features, contributing to the observed shifts in BE.
2. Excitation Effects: While theoretical BE values typically account only for electronic excitations, the experimental results possibly further include contributions from vibrational excitations of gas molecules besides electronic excitation. This discrepancy can result in additional peaks or shifts in the background of the experimental spectra that are not accounted for in theoretical predictions. Meanwhile, within one type of electronic excitation, there are still multiple transitions that occur with closely spaced transition energies. It's challenging to entirely resolve the details of every individual excitation energy within each excitation state. Thus, we used the electron transitions within the transition states to clarify the peak assignments to avoid misunderstanding or ambiguities.

**Revisions made**: (1) We have added Table R1 into the revised Supporting Information as Table S1 to compare the BE values obtained from our experiments with those reported in the literature.

(2) We have added the following discussion to the revised manuscript.
"IPES structures in the metal core-level spectra represent the scattering events with the highest probabilities, while the elevated background and spin-orbit splitting obscure the lower-probability scattering features, especially for $CO_2$ and $N_2$ (**Figure 1**, **Figure 2a** and **2e**). The individual IPES structures highlighted in **Figures 1** and **2** by solid colors were determined based on the BE values previously reported, which were used as the XPS curve-fitting constraints. This approach ensures that our assignments are guided by established reference results. The use of reported BEs as benchmarks provides a reliable foundation for identifying and deconvoluting these IPES structures. The BE values obtained from our experiments are comparable with those previously reported (Table S1), which demonstrates the consistency of our assignments and provides transparency regarding the alignments between our results and established references."

2. One of the more interesting questions in my mind is the nature of the excitations responsible for the IPES peaks. There is a brief comment of the assignments on p5 of the manuscript, which is not that easy to follow due to some typographical issues. I felt the assignments deserved a more careful explanation.

**Response:** We thank the Reviewer for highlighting this important aspect and pointing out the typographical issues in the initial manuscript. We have carefully revised the text to address these issues and improve the clarity of our explanations regarding the nature of the excitations

responsible for IPES. To provide a more comprehensive and precise illustration, we have included Table R1 in the revised Supporting Information as Table S1, which details the peak assignments based on excitation states within the atomic and molecular orbitals. These assignments are grounded in molecular orbital theory and relevant spectroscopic studies from the references in Table R1. In Table R1, we categorize the observed peaks by their corresponding excitation states, each of which involves groups of closely spaced excitation energies rather than individual transitions. This approach acknowledges the inherent challenge of resolving every individual excitation energy within a single excitation state due to the spectral broadening and overlapping of peaks. The use of excitation states allows for a more accurate and systematic representation of the observed features in the IPES phenomena. We hope this revised explanation and the inclusion of Table S1 will provide the Reviewer and readers with a clearer understanding of the nature of IPES structures while addressing the ambiguities in our initial discussion.

**Revisions made**: (1) We have added Table R1 into the revised Supporting Information as Table S1.

(2) We have corrected the discussion in the revised manuscript as follows.
"The distinctive energy losses of +11.2 eV and +13.1 eV beyond the $CO_2$ main peak correspond to the excitation states of $^1\Sigma_u^+$ (which is also likely to be $^3\Sigma_u^-$, $^1\Pi_g$ or $^{3,1}\Pi_u$), and the first ionization transition state of $CO_2^+$ $^2\Pi_g$, respectively[15-16,34-36]. In the case of $N_2$, the IPES structures at +13.0 eV, +14.1 eV, and +16.0 eV compared to the main peak of $N_2$ can be corelated to two excitation states of $^2\Pi_g$ and $^2\Sigma_g^+$[37-42], respectively. Additionally, several excitation states (C $^1\Pi_u$, B $^1\Sigma_g^+$, c $^3\Pi_u$, a $^3\Sigma_g^+$, E(F) $^1\Sigma_g^+$) within the $H_2$ molecular orbitals inform the IPES structures observed for $H_2$ molecules within the metal core-level regions[43-47]. The assignments of all IPES structures are summarized in Table S1. We categorize the observed peaks by their corresponding excitation states, each of which involves groups of closely spaced excitation energies rather than individual transitions. This approach acknowledges the inherent challenge of resolving every individual excitation energy within a single excitation state due to the spectral broadening and the overlapping of peaks."

3. There appears to be a problem with the text in some parts of the supplementary - perhaps this is a corrupted pdf file that needs fixing before publication.

**Response**: We thank the Reviewer for highlighting this typographical error. We have corrected the issues in the legends of Tables S2–S4 and ensured that they are now properly displayed.

**Revisions made:** We have corrected the display issues in the legends of Tables S2–S4 in the revised Supporting Information.

**We truly thank the reviewer for the insightful comments and kind suggestions! The reply for each question/comment is expected to reach the high criteria.**
